# A role for heritable transcriptomic variation in maize adaptation to temperate environments

**DOI:** 10.1186/s13059-023-02891-3

**Published:** 2023-03-24

**Authors:** Guangchao Sun, Huihui Yu, Peng Wang, Martha Lopez-Guerrero, Ravi V. Mural, Olivier N. Mizero, Marcin Grzybowski, Baoxing Song, Karin van Dijk, Daniel P. Schachtman, Chi Zhang, James C. Schnable

**Affiliations:** 1 Quantitative Life Sciences Initiative, University of Nebraska-Lincoln, Lincoln, USA; 2 Center for Plant Science Innovation, University of Nebraska-Lincoln, Lincoln, USA; 3 Department of Agronomy and Horticulture, University of Nebraska-Lincoln, Lincoln, USA; 4 School of Biological Sciences, University of Nebraska-Lincoln, Lincoln, USA; 5 Department of Biochemistry, University of Nebraska-Lincoln, Lincoln, USA; 6 Institute for Genomic Diversity, Cornell University, Ithaca, USA

**Keywords:** Expression quantitative loci, Maize transcriptional regulatory network, Temperate adaptation

## Abstract

**Background:**

Transcription bridges genetic information and phenotypes. Here, we evaluated how changes in transcriptional regulation enable maize (*Zea mays*), a crop originally domesticated in the tropics, to adapt to temperate environments.

**Result:**

We generated 572 unique RNA-seq datasets from the roots of 340 maize genotypes. Genes involved in core processes such as cell division, chromosome organization and cytoskeleton organization showed lower heritability of gene expression, while genes involved in anti-oxidation activity exhibited higher expression heritability. An expression genome-wide association study (eGWAS) identified 19,602 expression quantitative trait loci (eQTLs) associated with the expression of 11,444 genes. A GWAS for alternative splicing identified 49,897 splicing QTLs (sQTLs) for 7614 genes. Genes harboring both *cis*-eQTLs and *cis*-sQTLs in linkage disequilibrium were disproportionately likely to encode transcription factors or were annotated as responding to one or more stresses. Independent component analysis of gene expression data identified loci regulating co-expression modules involved in oxidation reduction, response to water deprivation, plastid biogenesis, protein biogenesis, and plant-pathogen interaction. Several genes involved in cell proliferation, flower development, DNA replication, and gene silencing showed lower gene expression variation explained by genetic factors between temperate and tropical maize lines. A GWAS of 27 previously published phenotypes identified several candidate genes overlapping with genomic intervals showing signatures of selection during adaptation to temperate environments.

**Conclusion:**

Our results illustrate how maize transcriptional regulatory networks enable changes in transcriptional regulation to adapt to temperate regions.

**Supplementary information:**

The online version contains supplementary material available at 10.1186/s13059-023-02891-3.

## Introduction

Whole organism phenotypes are determined by a combination of genetic and environmental factors. Selection scan methods can identify loci with different effects on fitness in natural populations adapted to different environments or different effects on traits deemed desirable by humans in domesticated species [1, 2]. However, these comparative population genetic approaches typically do not determine the mechanisms by which selected loci alter a given phenotype. Although there are numerous exceptions, genetic variants tend to act on phenotypes by changing the coding sequence and, thus, protein function or by affecting regulatory sequences for transcriptional control, ultimately leading to alterations in protein abundance. The potential effect size of DNA sequence variants on protein function can be predicted through multiple approaches informed by protein structure, amino acid similarity, and/or evolutionary conservation. Predicting the effect of DNA sequence variants on transcript abundance remains far more challenging, although variant partitioning suggests that 50–55% of variance for various phenotypes is explained by non-coding sequence features [3]. As transcript abundance can be profiled across many individuals, variants associated with variation in the abundance of individual mRNA transcripts can be empirically identified across the genome. Individuals carrying rare alleles in *cis*-regulatory regions for a given gene are disproportionately likely to exhibit gene expression levels in the extreme tails of population expression distribution [2]. Many of the earliest quantitative genetic studies of gene expression regulation were conducted in biparental populations of model organisms, including yeast (*Saccharomyces cerevisiae*) [4], Arabidopsis (*Arabidopsis thaliana*) [5], and maize (*Zea mays*) [6], from which two classes of loci were identified when using the transcript abundance of multiple genes (1) *cis*-acting regulatory variants mapping to the gene in question, playing a role in modulating the expression of a single gene, and (2) *trans*-acting regulatory variation mapping elsewhere in the genome, frequently at “hot-spots” [4–6]. Typically, variants acting in *cis* tend to explain more of the total variance in the expression of their target genes than *trans*-acting regulatory variations [5–7]. However, *trans* acting regulatory variants influencing the expression of multiple genes are frequently identified in single genomic intervals, thus forming “hot-spots” potentially corresponding to variation in a transcription factor or other regulators [4, 5, 8–10].

Mapping expression quantitative loci (eQTLs) using recombinant inbred line (RIL) has greater statistical power than biparental populations to identify variants with modest-sized effects mapping to regulatory hotspots associated with variation in the expression of many genes [7, 8, 11]. Since individual maize transcription factors bind to and regulate the expression of many genes [12], some or all hotspots may represent functional variants genes encoding transcription factors or other regulatory proteins. Importantly, RIL populations typically do not provide sufficient resolution to resolve mapping to individual candidate genes. By contrast, association mapping utilizing historical recombination events can achieve dramatically higher mapping resolution than biparental populations, particularly in maize where linkage disequilibrium (LD) decays much faster than in many other species [13], although this comes at a cost of requiring phenotypic data from more individuals [14]. Several eQTL studies using natural diversity panels have offered much higher resolution to map eQTLs to one or several candidate genes or regulatory sequences using transcriptome deep sequencing (RNA-seq). Examples include an analysis of a 224 line maize diversity panel under control and water stressed conditions [15] and an analysis at two stages of kernel development from 282 diverse maize lines [16, 17]. eQTL analyses in natural diversity panels frequently suffer from a lower power to discover the most of small to moderate effect variants segregating in the population, making it challenging to identify “hotspots” affecting the expression of multiple target genes.

Microarray-based measurements of gene expression were not specifically designed to distinguish between functionally distinct splice isoforms originating from the same gene. Similarly, 3′ mRNA sequencing provides a scalable and cost-effective mechanism to profile expression from large numbers of individuals but cannot quantify alternative splicing [18]. The expression of different splice isoforms has recently been shown to be regulated both in *cis*- and *trans* [19, 20]. Variation in alternative splicing has been reported to be associated with yield [21], development [22], stress tolerance [23, 24], and climate adaptation [25] in plants. As technology for quantifying transcript abundance has advanced, more studies have identified genetically controlled variation in gene expression linked to whole plant phenotypic diversity that contributes to local adaptation, as reviewed by Cubillos et al. [26]. For example, seed shattering in field mustard (*Brassica rapa*) [27] and domesticated rice (*Oryza sativa*) [28] exhibited diversity due to the same allelic variation in local regulatory elements of *REPLUMLESS* (*RPL*) and *Shattering 1* *qSH1*, respectively. Furthermore, apical dominance in maize which played a major role in domestication, is due to an upstream transposon insertion of the *Teosinte branched 1* (*TB1*) gene [29].

Maize is both a major crop and a model for plant genetics and genomics. After being domesticated 10,000 years ago in what is now south central Mexico [30], cultivated maize spread into the region that is now the southwestern United States. There, the expansion of maize cultivation range stalled for many years, likely as a result of the poor adaptation of tropical maize to the environments found in more temperate latitudes [31]. Once selection produced maize varieties able to thrive in temperate climates, the crop rapidly spread throughout North America, with maize now being cultivated in a wide range of temperate and tropical regions. Temperate maize differs from tropical maize in a range of phenotypes including flowering time and photoperiod sensitivity [32, 33], as well as tolerance to stresses such as cold, low soil nitrogen, and moisture content [34, 35]. By exploiting heterosis, Flint elite temperate maize lines have made for good founders of maize germplasms well adapted to high latitudes [1]. Candidate genes showing significant selective sweep signals in this heterotic pool showed different haplotype diversity between the Flint and Dent groups, with the Flint haplotype promoting early flowering time [1]. This phenotypic divergence is predominantly explained by loci with individual small effects [32]. However, a few large-effect loci have also been identified for natural variation in temperate adaptation traits including *Vegetative to generative transition 1 (Vgt1)*, a QTL that produces a 4–5-day change in flowering time and results from polymorphism in a regulatory sequence controlling the transcription of *Related to APETALA 2.7 (ZmRap2.7)* [36]. A study using a maize diversity panel including lines widely adapted to temperate environment in China and tropical lines from CIMMYT in Mexico identified genic regions related to flowering time, stress response, development, and metabolic processes might be associated with temperate adaptation [37]. However, the study used 558,529 SNPs called from RNAseq reads leaving out the genetic variants in non-coding regions where important regulatory elements for expression usually present [9, 29]. Another study conducted using resequencing data of 35 improved maize lines, 23 traditional landraces, and 17 wild relatives also identified genomic regions associated with maize domestication and improvements [38]. However, these studies either lack high density SNP markers covering the genic and nongenic regions which lead to loss of non-coding variants associated with adaptation in the case of Liu [37] or transcriptomic data to link the genomic features identified from selection analysis with the phenotypic variations in the case of Hufford [38]. In addition to genome wide selective sweep scanning attempting to discover genomic basis for environment adaptation or artificial selection, several important studies also sought to link genomic loci with the associated phenotypic variations by intermediate traits. For example, a metabolite-based genome-wide association mapping and identified candidate genes associated with metabolic traits in maize kernel [39]. Furthermore, comparison of epigenetics variation in maize landrace, modern maize and teosinte identified genomic regions exhibiting differential methylation patterns which are important for morphological change of maize during domestication and improvements [40].

Here, we profiled gene expression variation by RNA-seq across a 340-line maize diversity panel comprising a large subset of the Buckler-Goodman maize association panel [41], augmented with additional diverse maize inbreds. We generated and sequenced two or more biological replicates for most of these genotypes, allowing estimation of broad-sense heritability for each annotated and expressed maize gene. We then mapped both expression QTL (eQTLs) and splicing QTL (sQTLs) using a high density set of 12 million single nucleotide polymorphism (SNP) markers. Consistent with previous expression mapping efforts in association populations, our power to identify *trans*-regulatory hotspots via single gene analysis was limited. However, independent component analysis enabled the identification of latent features associated with the expression of multiple genes, making it possible both to identify genomic intervals controlling these latent transcriptional features and to assign putative functions to the latent features in processes including cell wall biogenesis, plant-pathogen interactions, fatty acid metabolism, and plant hormone biosynthesis. Using 40 unambiguous tropical genotypes and 52 unambiguous temperate genotypes, we identified a set of *cis*-regulatory elements and their targets associated with endosperm color, flowering time, and upper kernel shape. Importantly, these variants showed significant differences between tropical maize and temperate maize. We also showed that a set of genes with lower expression heritability in temperate maize are also enriched in functions essential for temperate adaptation such as vegetative to reproductive growth transition, cell cycle regulation, and gene silencing.

## Results

### Changes in the heritability of gene expression associated with adaptation and breeding to temperate climates

**Fig. 1:**
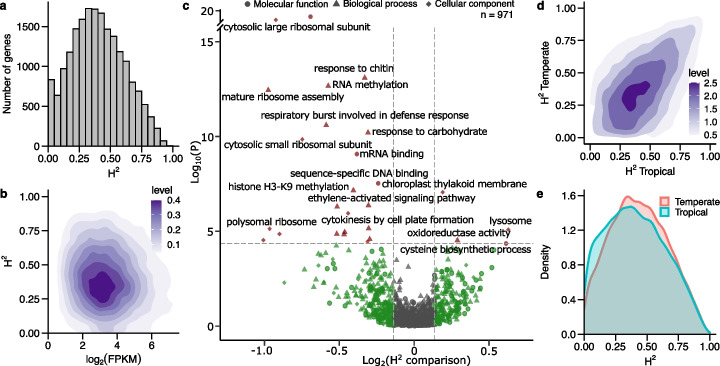
Broad sense heritability calculated from 19,565 genes among replicated maize lines, temperate panel and tropical panel. **a** Distribution of broad sense heritability (*H*^2^). **b** Two-dimensional scatter plot between heritability and Log_2_ of mean FPKM values across the population; level indicate the density of the data points. **c** Volcano plot of Gene Ontology (GO) terms that show significant distributional differences across the population. Statistical difference of the distribution between the *H*^2^ in study gene set and the remaining background set was conducted using the Kolmogorov-Smirnov test for goodness of fit. *p*-values are adjusted for a false discovery rate (FDR) $\le$ 0.05; a full list of GO terms with labels is provided in Additional file 2: Table S2. **d** Distribution of broad sense heritability of genes in the tropical and temperate panels; **e** Density of gene expression broad sense heritability in tropical (blue) and temperate (red) maize lines

We generated an average of 18 million sequence reads per sample from 572 RNA samples isolated from maize seedling roots grown under normal conditions (see the “Methods” section, Additional file 2: Table S1), consisting of 340 distinct maize genotypes, with 219 genotypes replicated two or more times. We identified 19,565 expressed genes to a level of 1 fragments per kilobase of transcript per million mapped reads (FPKM) or above in at least 458 (80%) samples. Employing the subset of genotypes with two or more replicates, we determined that the distribution of estimated broad sense heritability (*H*^2^; an estimate of total variance explained by differences between genotypes) is centered around 0.39 and shows an average value of 0.40 (Fig. 1a). We observed a modest and negative correlation (Spearman’s $\rho$ = − 0.09) between average expression and heritability for expressed genes (Fig. 1b). Estimates of the broad sense heritability of gene expression calculated from the expression of genetically identical plants grown at different times were reasonably well correlated with estimates of narrow sense heritability calculated from per-genotype average expression values (Additional file 1: Fig. S1 a), but with with a greater dynamic range observed in the case of broad sense heritability (Additional file 1: Fig. S1 b). As all genotypes employed in this study were inbreds, this difference may be explained by differences in the methods used for calculation of the two types of heritability, although it may to some extent also reflect the contribution of epistatic interactions to gene regulation. The genetic control of alternative splicing—using percent-spliced-in (PSI) values of introns as a readout—was also assayed. Unlike overall gene expression, a large proportion of introns exhibited almost no heritable variation in splicing, while a small fraction did exhibit substantial heritable variation in splicing (Additional file 1: Fig. S1 c &d). Among 971 non-redundant Gene Ontology (GO) terms assigned to between 25 and 499 expressed genes, genes annotated with six GO terms exhibited significantly higher (> 10% and one tail *t*-test *p* $\ge$ 0.05 ) median heritabilities than the median expression heritability of the overall set of expressed genes, while 23 non-redundant GO terms exhibited significantly lower (< 10%) median heritabilities (Fig. 1c; Additional file 3: Table S2). Genes annotated with GO terms linked to cysteine biosynthetic process, lysosome, anti-oxidation, and photosynthesis were among those whose expression in seedling roots tended to be more heritable across the diversity panel tested (Fig. 1c). Genes with lower expression heritability included those annotated as being involved in environmental or pathogen responsive activity as well as histone H3K9 methylation and protein kinases (Fig. 1c; Additional file 3: Table S2).

The panel profiled here included 40 genotypes of unambiguous tropical origin and 52 unambiguous temperate maize genotypes [42] (Additional file 4: Table S3). Estimates of gene expression heritability calculated from either the unambiguously tropical or unambiguously temperate populations were reasonably well correlated with each other (Fig. 1d). Mean of expression heritability in temperate panel (0.374) was modestly but statistically significantly higher than that of tropical panel (0.324) (*p* <2.2e−16; paired *t*-test) (Fig. 1e). In 1251 cases, the expression heritability of a given gene among temperate maize genotypes was less than 20% the value observed in the tropical maize lines. These 1251 genes exhibited significant enrichment for functional annotations linked to chromosome organization, nitrogen compound metabolism, epigenetic regulation of gene expression, cell proliferation, immune effector process, and regulation of flower development (Additional file 1: Fig. S2; Additional file 5: Table S4).

### Heritable transcriptomic variation captured by expression quantitative loci

We mapped expression quantitative trait loci (eQTLs) using MatrixEQTL [43], implementing an univariate linear model for the expression level of each of the 19,565 expressed maize genes using 12,191,984 SNPs generated from a combination of RNA-seq based SNP calling and data from the maize HapMap3 project (see the “Methods” section) [44].

**Fig. 2:**
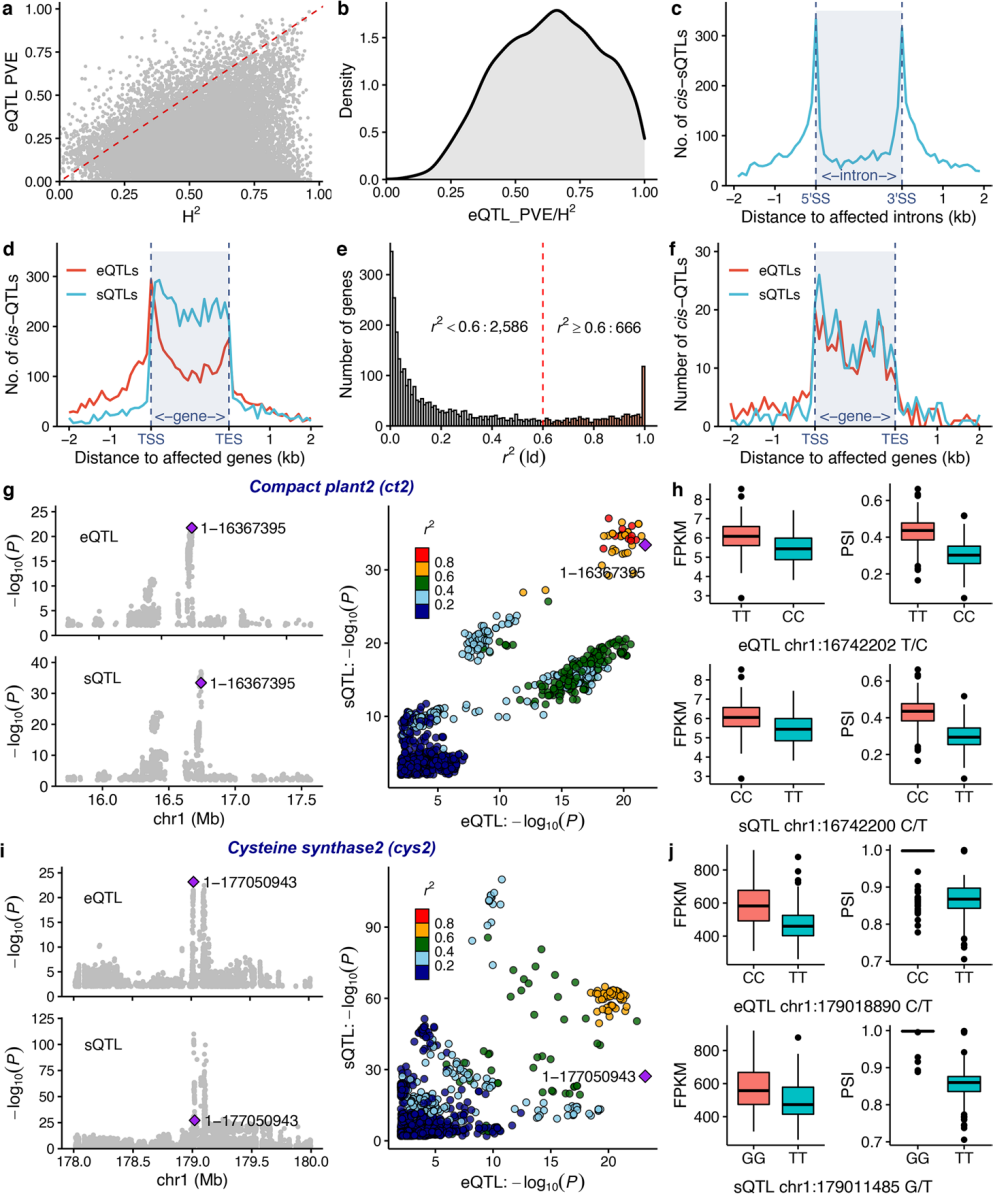
Expression and splicing quantitative trait loci (QTLs) and their relations. **a** Relationship between expression heritability (*H*^2^) and cumulative percentage of expression variation explained (PVE) for all eQTLs identified for a given gene. **b** Proportion of expression heritability (*H*^2^) explained by eQTLs. **c** Rapid linkage disequilibrium (LD) decay in the maize genome allows a highly resolved location for *cis*-sQTLs relative to their targets. 5ŚS : 5 prime splicing site; 3ŚS : 3 prime splicing site. **d** *cis*-expression QTLs (*cis*-eQTLs) are enriched at transcription start and stop sites, while *cis*-splicing QTLs (*cis*-sQTLs) tend to reside in gene bodies. TSS, transcription start site; TES, transcription end site. **e** LD r^2^ distribution of the *cis*-eQTLs and *cis*-sQTLs identified for the same genes. **f** Co-localization of highly linked (*r*^2^ > 0.6) *cis*-eQTLs and *cis*-sQTLs relative to their target genes. **g** *COMPACT PLANT2* (*ct2*) is co-regulated by *cis-*regulatory elements affecting both gene expression levels (eQTL) and splicing variation (sQTL). LD *r*^2^ is color coded and the lead SNP of eQTL (SNP name: 1-16367395, located at chr1:16,742,202) is highlighted as a purple diamond in the right panel. **h** Box plots of gene-level expression (FPKM) and splicing variation of the target intron (PSI) in *ct2* in maize lines carrying different alleles of the lead SNP representing eQTL (chr1:16,742,202 T/C) (top panel) and the lead SNP representing the sQTL (chr1:16,742,200 C/T) (bottom panel). **i** *Cysteine synthase 2* (*cys2*) is associated with both a *cis*-eQTL and a *cis*-sQTL. LD *r*^2^ is color coded and the lead SNP of eQTL (SNP name: 1-177050943, located at chr1:179,018,890) is highlighted as a purple diamond in the right panel. The two lead SNPs of eQTL and sQTL are not highly linked, indicating that the two regulatory elements may function independently. **j** Box plots of gene level expression (FPKM) and splicing-variation of the target intron (PSI) in *cys2* in maize lines carrying different alleles of the lead SNP representing eQTL (chr1:179,018,890 C/T) (top panel) and the lead SNP representing the sQTL (chr1:179,011,485 G/T) (bottom panel)

We identified 11,444 genes with 1 to 10 independent eQTL peaks (these genes are referred to as e-traits below). Among the 7691 e-traits with single discrete eQTL peaks, 7015 (92.4 %) are e-traits with *cis*-eQTLs, and the remaining 586 (7.6 %) are e-traits with only *trans*-eQTLs (Additional file 1: Fig. S3a). The small minority (161 e-traits) for which more than 10 distinct peaks were identified were excluded from downstream analyses, as manual examination of GWAS results for a subset of e-traits for which more than 10 distinct peaks were identified frequently revealed strong and scattered signals across the genome inconsistent with accurate identification of causal loci (Additional file 1: Fig. S3b). Considering e-traits with two or more identified eQTLs (but, as a result of the peak grouping strategy employed here a maximum of one possible detected *cis*-eQTL peak per e-trait), the overall breakdown was 54.2% of *cis*-eQTLs and 45.8% of *trans*-eQTLs, and for a set of 3521 e-traits with *cis*-eQTLs, *trans*-eQTLs were also detected (Additional file 1: Fig. S3c). Given the limited size of this mapping population and that many *trans*-eQTLs exhibit minor allele frequencies near 0.1, our power to detect *trans*-eQTL was necessarily limited and so the total number of true *trans* regulatory variants is likely substantially higher than the number of detected *trans* regulatory variants. Likely as a consequence, we observed no obvious *trans*-eQTL hotspots, in contrast to a previous eQTL mapping study conducted in maize using a bi-parental RIL population [8] where both alleles are of functionally distinct regulatory variants are typically present in approximately 50% of individuals (Additional file 1: Fig. S3d). Consistent with expectations, the cumulative percentage of expression variation explained by all eQTLs (PVE) identified for a given e-trait was typically lower than the estimate of total variance explained from genetic factors (e.g., expression heritability) (Fig. 2b). It should be noted that percent variance explained values presented in this study were calculated from the same dataset used to identify eQTLs. As a result, these values are likely to be inflated to some extent relative to the percent variance explained that would be observed for the same eQTLs in an independent dataset.

*cis*-eQTLs tended to play larger roles in explaining total expression variance for the associated e-trait than *trans*-eQTLs (Additional file 1: Fig. S3e). Discovered *trans*-eQTLs were significantly more likely to represent rare alleles in this maize population (defined here as alleles with a minor allele frequency of <0.2) than *cis*-eQTLs (Additional file 1: Fig. S3f). Among the 8984 *trans*-eQTLs identified in this study, 3662 have minor allele frequency lower or equal to 0.1, while the remaining 5322 have minor allele frequency higher than 0.1 (Additional file 1: Fig. S3f). A permutation based approach was used to evaluate whether the *p*-value threshold for eQTL is stringent enough to control potential false associations caused by population structure (see Methods). The *p* value cutoff that would correspond to a 5% chance of false discovery for any given gene was found to be 10^−7.83^ which is less stringent than the post Bonferonni correction *p*-value of 10^−8.39^ employed in this study (Additional file 1: Fig. S3g). Give a *p*-value of 10^−8.39^, the results of permutation testing suggest a false discovery rate of 0.015 for *trans*-eQTLs. Furthermore, a set of 3000 genes were randomly selected for parallel analyses using the mixed linear model implemented in GEMMA [45] which includes a control for kinship in addition to population structure. A similar proportion of genes with eQTL and similar numbers of eQTL per gene were observed in the GEMMA analysis (Additional file 1: Fig. S4a). However, GEMMA identified 20–30% fewer eQTL with minor allele frequencies < 0.1, suggesting reported eQTL with minor allele frequencies in this range are more likely to represent false positives than reported eQTL with higher minor allele frequencies (Additional file 1: Fig. S4b). Overall patterns reported by the two algorithms were similar and most peaks detected by MatrixEQTL were also visible in GEMMA Manhattan plots but sometimes below the threshold of statistical significance in the second analysis (Additional file 1: Fig. S4c).

### Alternative splicing QTLs are co-opted with eQTLs for transcriptional regulation

Using a previously published canonical PSI value based method (see the “Methods” section) to quantify alternative splicing events for introns, we performed splicing quantitative trait loci (sQTLs) mapping using same approach as eQTL mapping, peak grouping, and filtering. 49,897 sQTLs for 16,437 s-traits in 7614 annotated genes were identified (Additional file 1: Fig. S5a). We detected more *trans*-sQTLs than *cis*-sQTLs (Additional file 1: Fig. S5b), while *cis*-sQTLs tended to have larger effects and explained a larger proportion of splicing variation than detected *trans*-sQTLs (Additional file 1: Fig. S5c), in agreement with a previous report employing gene expression data from developing maize kernels [20]. Of the s-traits evaluated, 33.5% are under both *cis*- and *trans*-regulation (Additional file 1: Fig. S3d). Again, this is very likely a dramatic underestimate of the true number of segregating variants given the low power to detect *trans-* regulatory elements with modest minor allele frequencies in this population. We resolved the distance between *cis*-sQTLs and their targets within 1 kb, suggesting that the rapid decay of LD in this maize population enables high resolution mapping of sQTL locations (Fig. 2c).

In contrast, to the pattern observed for *cis*-eQTLs which we frequently observed at the annotated transcription start sites (TSSs) and transcription end sites (TESs) of their associated genes, *cis*-sQTLs were highly enriched in the gene body, particularly at the splicing sites of introns (Fig. 2d).

In 3252 cases, we determined that an individual maize gene is associated with both a *cis*-eQTL and at least one *cis*-sQTL (Fig. 2e). However, in many cases the peak SNPs of the *cis*-eQTL and the *cis*-sQTL were not in LD with each other. To reveal the potential colocalization between *cis*-eQTLs and *cis*-sQTLs, we calculated the R-squared values (*r*^*2*^) for LD between the peak SNP for the *cis*-eQTL and *cis*-sQTL for the same gene, resulting in the identification of 666 genes with colocalizing *cis*-eQTL and *cis*-sQTL peaks (*r*^*2*^ $\ge$ 0.6) (Fig. 2e). The density of *cis*-eQTLs and *cis*-sQTLs was similar for these 666 genes in gene bodies (Fig. 2f). Among different types of splicing variations, 358 out of these 666 genes exhibited variation in intron retention rates, and their expression levels are highly correlated with the splicing level (PSI) of the introns (with a false discovery rate [FDR] < 0.05). However, the direction of correlation was not consistent. Indeed, 226 out of these 358 cases exhibited a positive correlation with their expression levels, while the remaining 132 cases showed a negative correlation (chi-square test *p* = 6.76e^−7^). This result was consistent with a previous report of the “intron-mediated enhancement” phenomenon whereby the number of introns in a gene tends to be positively associated with expression levels [46]. We provide two examples to demonstrate cases where expression and splicing are jointly regulated or independently regulated within the same gene, using visualizations from the locuscomparer package [47]. Mutations in *COMPACT PLANT2* (*ct2*; *Zm00001d027886*) are associated with shorter plant height and wider meristems, leaves, and ears; *ct2* encodes a G$\alpha$ subunit of a heterotrimeric GTP-binding protein [48]. In the population analyzed here, both overall *ct2* expression and alternative splicing of the first intron (within the 5′ untranslated region) were controlled by one linked *cis*-eQTL and one *cis*-sQTL (*r*^*2*^ = 0.86) (Fig. 2g). The highly linked lead SNPs representing *cis*-eQTL and *cis*-sQTL of *ct2* exhibited near identical effect to both gene level expression and splicing of the target intron in *ct2* (Fig. 2h). *cys2 *(Zm00001d031136) expression level was associated with a *cis*-eQTL located in the *cys2* promoter region, while a *cis*-sQTL within the gene body modulated the retention level of the first intron in the 5’ UTR. Despite being located only 7.4 kb apart (Fig. 2i), the *cis*-eQTL and *cis*-sQTL located within *cys2* have a disequilibrium *r*^*2*^ of 0.33 suggesting that these two SNP markers may define separate functional variants controlling expression levels and transcript splicing (Fig. 2j).

### Independent component analysis revealed heritable latent *trans-* regulatory hubs for co-expression modules

**Fig. 3:**
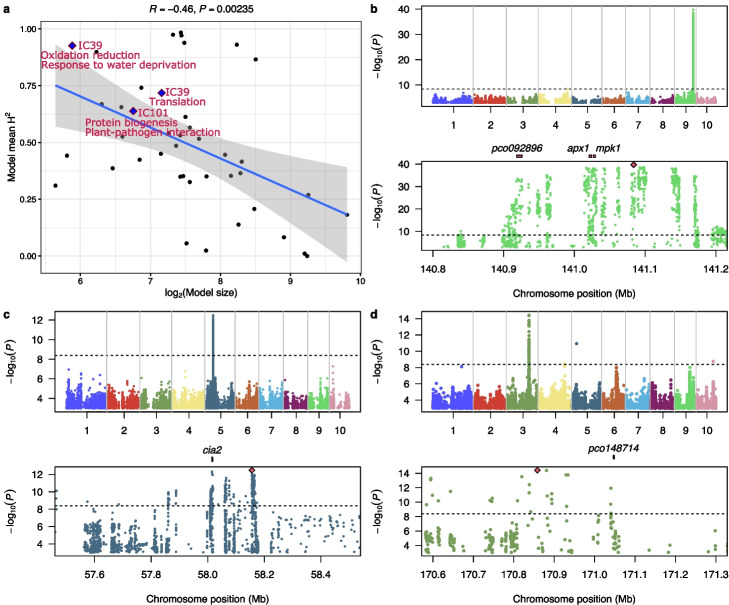
Independent component analysis identified co-expression modules related to important growth and stress response related pathways. **a** The correlation between heritability of independent components and the number of their associated co-expression gene modules. IC39, IC79 and IC101 and the function of the co-expression gene modules enriched were labeled under the independent component IDs. The correlation coefficient (*(*R)) and *p*-value (*(*P)) are shown on the top. **b** Genome wide association study (GWAS) of IC39 (top panel) and three candidate genes (*pco092896* (Zm00001d047755), *apx1* (Zm00001d047757), and *mpk1* (Zm00001d047758)) identified within the regions exhibiting significant signals on chromosome 9 (bottom panel). **c** Genome wide association study (GWAS) of IC79 (top panel) and one candidate gene (*cia2* (Zm00001d014664)) identified within the regions exhibiting significant signals on chromosome 5 (bottom panel). **d** Genome wide association study (GWAS) of IC101 (top panel) and one candidate gene (*pco148714* (Zm00001d042533)) identified within the regions exhibiting significant signals on chromosome 5 (bottom panel). In **b**, **c**, and **d**, the lead SNPs are highlighted and the dashed horizontal lines indicate the *p*-value threshold

eQTL analysis in natural diversity populations frequently lacks the power to discover most of small to moderate effect variants making it challenging to identify regulatory hotspots. Using the method proposed by Rotival et al. [49], we employed independent component analysis of the expression matrix (572 RNA-seq datasets × 19,565 expressed gene models) to generate a signature matrix of latent features reflected in the expression patterns of multiple genes. A set of 166 independent components explained approximately 80% of the total variance of the expression matrix (Additional file 1: Fig. S6). After filtering independent components based on module distribution kurtosis [50], we retained 42 independent components associated with the expression of 24 to 720 genes for downstream analysis (see the “Methods” section). Heritability of these 42 independent components varied from 0 to 0.98 and together explained 34.15% of the expression variance in the population (Fig. 3a). Moreover, the heritability of the 42 independent components tended to show a negative correlation with the number of genes included in the co-expression module associated with the independent components (Fig. 3a). This observation suggests the latent features identified by this method might represent *trans*-eQTL hubs composed of a large number of small effect regulatory genomic loci. If so, these may explain a portion of heritability not captured by traditional eQTL mapping. GO enrichment analysis revealed that the co-expression gene hub associated with IC39 tend to be involved in response to oxidative stress and water deprivation stress; co-expression gene hub associated with IC79 tend to be enriched in protein translation; co-expression gene hub associated with IC101 are enriched in protein folding and plant-pathogen interaction (Fig. 3a).

We conducted genome-wide association study for each of the 42 independent components, followed by grouping of individually significant trait associated SNPs into distinct peaks as described for peak grouping for eQTL and sQTL mapping (see the “Methods” section). Expression QTL mapping was conducted between the SNPs in IC GWAS peaks and expression of the 19,565 using a relaxed significant threshold (10e−5) for eQTL identification [49]. A peak represented by the lead SNP located at 141 Mb on chromosome 9 (9:141,083,854) was associated with expression variation of 26 out of 59 genes included in IC39 module. This lead SNP was in high linkage disequilibrium with Zm00001d047757 encoding *apx1*, Zm00001d047755 (*pco092896*) encoding agrogenate dehydratase 2 chloroplastic, a homolog of Arabidopsis ADT2 [51], and Zm00001d047758 encoding *mpk1*. *apx1* has been shown to be involved in response to heat, drought stress [52], and reactive oxygen species (ROS)-scavenging [53] in Arabidopsis. ADT2 has been shown to be involved in seed development and *mpk1* is also mapped within a QTL associated with leaf stomatal activity in response to drought in maize [54]. Functions of these three candidate genes were in agreement with the functions that are enriched in the module (Fig. 3b). For IC79, the lead SNP was located at 58.1 Mb on chromosome 5 (5:58,155,918) where a gene nearby (Zm00001d014664) encodes Protein CHLOROPLAST IMPORT APPARATUS 2, a homolog of the Arabidopsis CIA2 that upregulates expression of translocons Toc33 and Toc75 in leaves, which are essential for protein import into chloroplasts [55, 56]. This is consistent with the functions enriched in the module genes associated with IC79 (Fig. 3c). GWAS of IC101 identified a strong signal on chromosome 3 (Fig. 3d); the lead SNP of the peak located at 170.8 Mb (3:170,858,379) is nearby Zm00001d042533 encoding a trigger factor, which is responsible for plastidic protein biogenesis and folding in green algae and land plant [57]. In addition, the e-traits of this peak identified by GWAS included many genes associated with plant pathogen responsive genes such as Zm00001d022517 encoding a NAC transcription factor, Zm00001d023326 encoding rust resistance-like protein (*rp1-4*), and Zm00001d023316 encoding a disease resistance protein (*rpm1*). These observations are consistent with the functions enriched in the module associated with IC101 (Fig. 3d). The alignment of the enriched function of genes whose expression was directly linked to the locus and the enriched functions among genes associated with the IC is not surprising would not necessarily be true in all cases and the expression of only a subset of genes associated IC were directly linked to the peak and the expression of some genes not in the IC were also directly linked to the peak. Moreover, among the five candidate genes mentioned above, four of them, except for *cia2*, showed significant reduction in expression heritability in temperate maize panel (Additional file 1: Fig. S7a). The average expression of genes in IC39 (Additional file 1: Fig. S7b) and IC79 (Additional file 1: Fig. S7c) and IC101 (Additional file 1: Fig. S7d) were reasonably consistent between tropical and temperate maize populations, although several individual exhibit large drops in IC39 and IC79.

### Genomic regions associated with temperate adaptation in maize

**Fig. 4:**
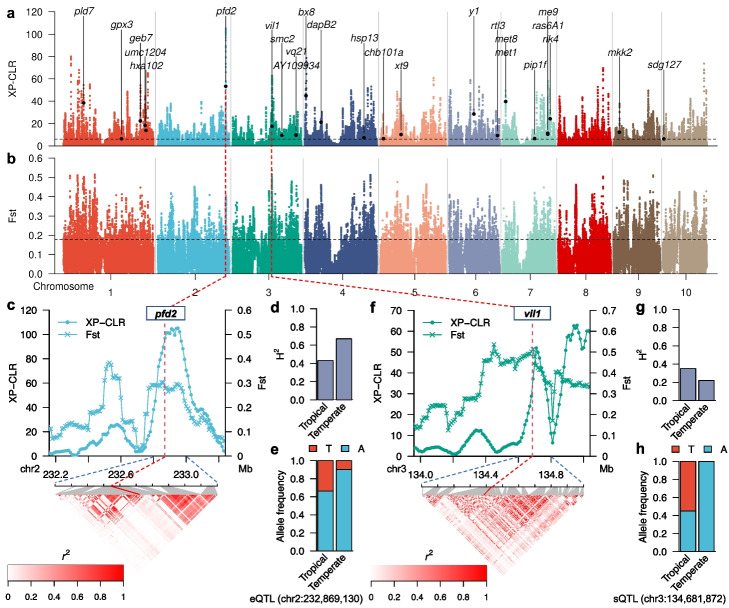
Genome-wide analysis of selective sweeps for temperate adaptation. **a** Genome-wide XP-CLR scanning in 100-kb sliding windows with a 10-kb step size. The dashed horizontal line indicates the top 10% XP-CLR threshold. Characterized genes with significant selective sweep signals that also showed reduction of expression heritability in temperate maize (except for *pfd2*) are indicated. **b** Genome-wide fixation index (Fst) scanning in 100-kb sliding windows with a 10-kb step size. The dashed horizontal line indicates the top 10% Fst threshold. **c** The maize homolog of Arabidopsis *PREFOLDIN-2* (*pfd2*) located near the highest XP-CLR peak. Red dashed line indicates the position shown in **a** and **b**; blue dashed lines below the *x*-axis indicates the boundary of the regions in which linkage disequilibrium *r*_2_ was analyzed; the heatmap indicates LD *r*_2_. **d** Difference in *pfd2* expression heritability between temperate and tropical maize. **e** Frequency of different alleles at the lead SNP (chr2:232,869,130 T/A) representing the eQTL of *pfd2* in tropical and temperate maize. **f** *VIN3-LIKE PROTEIN-1* (*vil1*) located near the highest Fst peak. Red dashed line indicates the position shown in **a** and **b**; blue dashed lines below the *x*-axis indicates the boundary of the regions in which linkage disequilibrium *r*_2_ was analyzed; the heatmap indicates LD *r*_2_. **g** Difference in *vil1* expression heritability between temperate and tropical maize. **h** Frequency of different alleles at the lead SNP (chr3:134,681,872 T/A) representing the sQTL of *vil1* in tropical and temperate maize

To identify genomic regions exhibiting significant selective sweep signals for adaptation to temperate environment, we performed cross-population composite likelihood ratio (XP-CLR) and fix index (Fst) scan in 100-kb windows with a 10-kb sliding window (see the “Methods” section). We obtained 2503 annotated maize genes located within 100-kb genomic intervals above the 90th percentile for both observed haplotype frequency difference (XP-CLR) and fix index (Fst) in a comparison of 40 unambiguous tropical and 52 temperate maize genotypes included in this study (Fig. 4a, b). A very limited overlap between two previously studies that conducted similar analysis within different populations [37, 38] likely due to population composition and genome assembly differences (Additional file 1: Fig. S8)

The highest XP-CLR value identified in this scan was for a genomic interval encompassing a maize homolog of *Arabidopsis* *PREFOLDIN SUBUNIT-2* (*pfd2*, Zm00001d007490) (Fig. 4c). This gene exhibited a slight change in expression heritability between tropical and temperate populations (Fig. 4d) and a modest change in allele frequency of a peak SNP associated with the *cis*-eQTL detected for this gene (Fig. 4e). The highest Fst value identified was for a genomic interval comprising a maize homolog of Arabidopsis *VERNALIZATION-INSENSITIVE-LIKE PROTEIN-1* (*vil*, Zm00001d041715) (Fig. 4f). This gene showed a large decrease in expression heritability between tropical and temperate populations (Fig. 4g), as well as a large shift in allele frequency for the *cis*-sQTL associated with splicing (Fig. 4h).

Of the 1058 potential target genes of selective sweeps between tropical and temperate maize germplasm that are associated with *cis*-eQTLs or *cis*-sQTLs in maize roots 171 showed a greater than 20% reduction in estimated heritability of gene expression between tropical and temperate lines including *Yellow 1* (*y1*, Zm00001d036345) [58], a target of artificial selection in temperate maize lines; *vil1* [59], the basic helix-loop-helix (bHLH) transcription factor gene *zmbhlh125* (Zm00001d045212) [60]; as well as additional genes potentially involved in temperate adaptation based on either functional characterization or functional prediction (Additional file 6: Table S5).

### Involvement of transcriptional regulatory elements in phenotypic adaptation in temperate maize

**Fig. 5:**
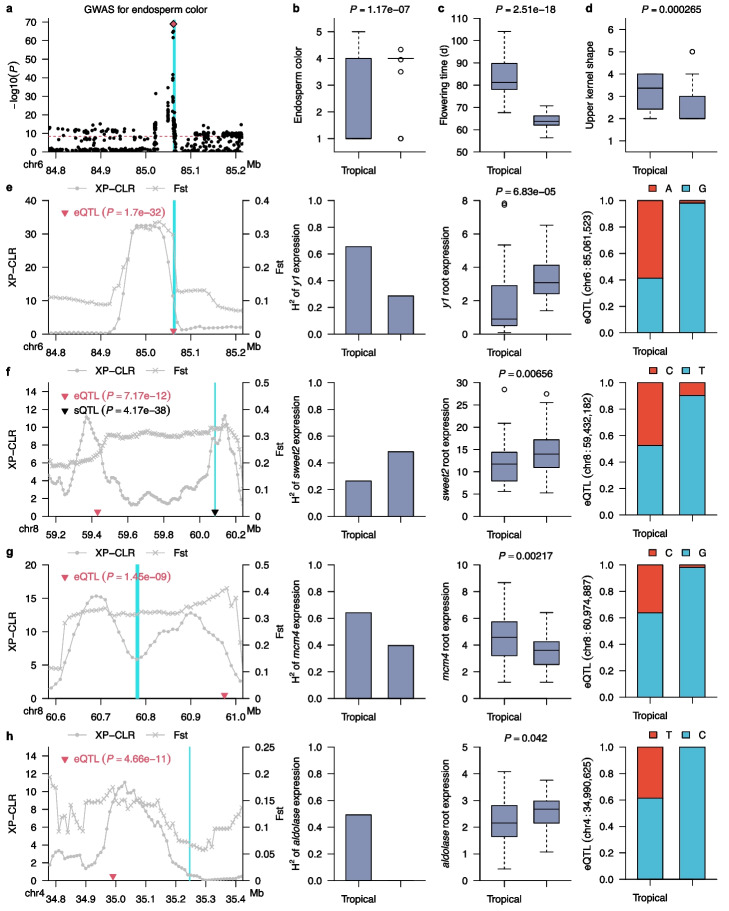
Candidate genes associated with selective sweeps in temperate maize. **a** Zoom in on the results of a GWAS for endosperm color using the maize genotypes included in this study focused on a 400 kilobase region containing the *y1* gene. The dashed horizontal line indicates a 0.05 Bonferroni corrected *p*-value threshold. Each gray dot indicates a SNP in this interval and the red dot indicates the peak SNP. Blue column on the left indicates the position of the *y1* gene. Lead SNP at position chr6:85,061,523 was highlighted as a purple diamond. **b** Difference in the distribution of endosperm color scores between temperate maize and tropical maize. This trait was quantified using the code: 1, white; 2, pale; 3, pale yellow; 4, yellow; 5, orange. **c** Difference in the distribution of flowering time (growing degree index from planting to 50% of anthesis) between temperate maize and tropical maize. **d** Difference in the distribution of upper kernel shape between temperate maize and tropical maize. This trait trait is quantified using the code: 1, shrunken; 2, indented; 3, level; 4, rounded; 5, pointed; 6, strongly pointed. **e** From left to right, selective sweep signals (XP-CLR and Fst), broad sense heritability of gene expression (*H*_2_) in tropical and temperate maize, average expression level (in FPKM) in the roots of tropical and temperate maize and the allele frequency of the peak SNP of the *cis*-eQTL in both tropical and temperate maize for *y1*. Blue column on the left indicates the position of the *y1* gene. **f** Display following the conventions of panel (**e**) for the *sweet2* gene. **g** Display following the conventions of panel (**e**) for the *mcm4* gene. **h** Display following the conventions of panel (**e**) for the *aldolase* gene

We identified one or more significant trait-associated SNPs in a genome-wide analysis using data from 14 of 27 previously published organismal-level phenotypes related to flowering, development, disease susceptibility, and yield (Additional file 7: Table S6), including the previously validated *y1* for endosperm color (Fig. 5a, Additional file 1: Fig. S9). After consolidating trait-associated SNPs into distinct peaks, we determined that candidate genes associated with organismal-level phenotypes and *cis*-regulatory elements with selection features for temperate adaptation such as reduced expression heritability in temperate maize or selective sweep signatures included candidate genes are potentially associated with endosperm color (Fig. 5a, b), flowering time (Fig. 5c and Additional file 1: Fig. S9), and kernel development (Fig. 5d and Additional file 1: Fig. S9). *y1* was previously reported to control maize endosperm color; we also identified *y1* in this study as exhibiting both a selective sweep signature (Fig. 5e; *first panel*) and reduced expression heritability (Fig. 5e; *second panel*). *y1* expression was significantly higher (Fig. 5e; *third panel*) in temperate maize and temperate maize inbreds predominantly carried the *G* allele at the *cis*-eQTL peak (Fig. 5e; *fourth panel*). *Sugars will eventually be exported 2* (*sweet2*, Zm00001d009365) was identified as a candidate gene associated with flowering time in this study (Additional file 8: Table S7) and exhibited selective sweep signatures (Fig. 5f; *first panel*) with a slightly increased expression heritability in temperate maize (Fig. 5f; *second panel*). The *cis*-eQTLs and *cis*-sQTLs for *sweet2* corresponded to two distinct XP-CLR peaks (Fig. 5f; *first panel*). *sweet2* expression was significantly higher in temperate maize relative to tropical maize (Fig. 5f; *third panel*) while the *T* allele was less common in the temperate maize lines included in this study (Fig. 5f; *fourth panel*).

We also identified the DNA replication licensing factor gene *mcm4* (Zm00001d009374) as a candidate gene associated with flowering time whose gene region and eQTL were nearby XP-CLR peaks (Fig. 5g; *first panel*). The expression heritability of *mcm4* was reduced by almost 50% in temperate maize compared to tropical maize (Fig. 5g; *second panel*), while its median expression level decreased by 25% in temperate maize (Fig. 5g; *third panel*). The *G* allele at the peak SNP for the associated *cis*-eQTL was much less common among temperate maize lines (Fig. 5g; *fourth panel*). *mcm4* is a DNA helicase that forms a ring-shaped MCM complex along with other MCM subunits to activate DNA replication origin sites, followed by unwinding the DNA helix and formation of the DNA-replication fork [61–63]. After DNA replication is initiated, the MCM complex is released and prevented from reloading onto the nascent DNA [64, 65]. The MCM complex is enriched in flowering bud tissue in Arabidopsis [63]. The *zmmcm4* gene is also stably expressed in maize flowering tissues (Additional file 1: Fig. S10 [66]. A gene encoding an aldolase superfamily protein (Zm00001d049559) was associated with upper kernel shape, with a *cis*-eQTL associated with its expression located within peaks for both XP-CLR and Fst (Fig. 5h; *first panel*). The heritability of expression for *aldolase* was much lower in temperate maize than in tropical maize (Fig. 5h; *second panel*) and exhibited a modest but significantly higher expression in temperate maize (Fig. 5h; *third panel*). In agreement with the selective sweep signature of the *cis*-eQTL, the allele frequency for the peak SNP of this *cis*-eQTL differs substantially between tropical and temperate lines (Fig. 5h; *fourth panel*). In addition, one of the SNPs linked to this gene was previously identified as a *cis*-eQTL at *Discolored 1* (*dsc1*, Zm00001d049872), which is associated with kernel development [67]. Both gene body and this eQTL mapped either within or nearby local Fst and XP-CLR peaks (Additional file 1: Fig. S11). *dsc1* exhibited significantly lower expression heritability in temperate maize compared to tropical maize, suggesting strong selection signals acting on its expression (Additional file 1: Fig. S11).

## Discussion

The regulation of transcription and mRNA abundance is a critical intermediate step mediating how the genotype determines the phenotype and phenotypic plasticity in response to environmental variation. However, molecular phenotypes such as the abundance of individual transcripts are subject to differing degrees of genetic and non-genetic control, as are whole organism phenotypes. Here, we generated partially replicated data from a panel of maize lines originating from different parts of the globe. We estimated the extend of variation in transcript abundance explained by genetic factors with single-gene resolution by including biological replication of genetically identical individuals. In addition, by employing random fragmentation of cDNA molecules rather than targeted 3′ end sequencing of mRNAs, we quantified the fraction of variation in mRNA splicing that is genetically controlled.

We identified 1251 genes with significantly lower broad sense heritability for their expression in temperate maize compared to tropical lines, indicating a transition to either become housekeeping genes or to be more environmentally responsive. The functions enriched in these genes included regulation of cell cycle and DNA methylation, vegetative to reproductive phase transition and transcription (Fig. 1). These results suggested that the genetic regulation of gene expression, cell propagation, and reproductive phase transition are likely power houses driving adaptation to temperate environments from tropical maizes inbreds to temperate inbreds. It should also be noted that this shift of expression heritability can be a result of genetic drift or selection which eventually lead to decrease of functional variants (Vg), therefore leading to decrease in broad sense heritability even though the environmental responsiveness is unchanged (Ve). In addition, the profiling of broad sense heritability by sequencing biological replicates allowed an estimation of all genetic factors (additive and non-additive) that explained variation in expression, in contrast to narrow sense heritability, which only measures additive genetic factors (Additional file 1: Fig. S1) [68–71]. The inclusion of non-additive genetic factors is not trivial because non additive factors such as dominance and epistasis explain a significant fraction of gene expression variation in both humans and plants [71, 72].

Alternative splicing is a complex regulatory process involved in co-transcriptional and post-transcriptional regulatory mechanisms [73–75]. Variational transcription can be reflected by mRNA levels (whole gene expression) controlled by eQTLs and by the ratio of transcript isoforms mediated by splicing-QTLs (sQTLs). The RNA-seq strategy used in this study captured sequence data from all portions of transcripts rather than solely at the their 3′ regions, allowing the quantification of splicing variation across samples and genotypes. The eQTLs and sQTLs identified in this study did not capture all expression heritability due to the limited size of the population, which limited the statistical power to detect low-effect *trans*-eQTLs or *trans*-sQTLs (Fig. 2a & b, Additional file 1: Fig. S5c). We conducted independent component analysis (ICA) [49] and determined that the components are heritable with broad sense heritability ranging from zero to one (Fig. 3a), suggesting that these heritable components might represent certain co-expression modules that together exhibit detectable heritability when background noise from other heritable modules is removed. In humans, ICA has detected broad impact eQTLs when confounding factors within the expression matrix reduces the power of eQTL detection [76]. GWASs between the SNPs and these components also identified genomic regions harboring genes with global regulatory functions. Distinct significant signals identified by GWAS for IC39, IC79, and IC101 revealed nearby candidate genes including *apx1*, *mpk1*, *cia2* and *pco148714* (Trigger factor). These genes are all characterized in Arabidopsis or green algae and their functions are consistent with the functional categories enriched in their associated co-expression gene modules (Fig. 3). We also noted a reduction in the estimated heritability the expression of these candidate genes in temperate maize panel. The co-expression gene modules these genes appear to control showed striking difference in certain genes (Additional file 1: Fig. S7).

In humans, spliceosome assembly co-opts RNA polymerase II for mRNA biosynthesis. Abolished spliceosome recruitment due to the artificial removal of introns leads to unprocessed RNA molecules that remain associated with RNA polymerase II, which eventually pauses on the nascent but unprocessed transcript [77]. The set of 666 genes with highly linked (co-localized) *cis*-eQTLs and sQTLs identified in this study may in fact support the possibility that a similar mechanism is at play in maize to regulate gene expression by eQTLs and sQTLs simultaneously (Fig. 2e–g). Furthermore, we also noticed that 33 of the 78 genes with annotations encoded transcription factors and a positive correlation between expression levels and degree of splicing was observed in a a significant portion of genes with intron retention variations would suggest a potential collaborative mode of action between transcription and splicing to achieve rapid response to environmental signals.

Maize domestication has reshaped the transcriptome; the genes showing differential expression patterns were shown to be involved in biotic stress responses compared to teosinte (*Zea mays ssp. parvigliumis*) [78]. A significant portion of the genes previously identified to be targets for domestication and evolution by population genetics also exhibited altered expression patterns [78]. Temperate adaptation is a major part of maize domestication and largely contributed to the current global distribution of this crop. Liu et al. [37] attempted to dissect the genetic architecture of temperate adaptation of maize at both genomic and transcriptomic level, a set of 2700 differentially expressed genes involved in stress adaptation between temperate and tropical-subtropical maize lines [37]. Temperate regions constantly impose drought stress onto maize during the growing seasons [33]. Wang et al. [79] showed that a 366 bp insertion in the promoter of *ZmVPP1*, encoding a vacuolar-type H^+^ pyrophosphatase, controls the drought inducible expression of *ZmVPP1* to confer drought tolerance in maize [79]. In this study, we systematically investigated the involvement of expression regulatory elements in phenotypes associated with temperate adaptation, using a combination of genome wide eQTL and sQTL mapping, genome wide selective sweep detection among temperate and tropical maize subgroups included in the RNA-seq population, and GWASs with phenotypes important for adaptation to temperate environments.

A set of 2503 genes mapped within regions enriched for selective sweep signals, several of which are promising candidates for the phenotypic adaptation of temperate maize. For example, the maize homolog of Arabidopsis *VIL1* was previously shown to be associated with flowering time and showed the highest XP-CLR value of all tested genes [59, 80]. Flowering time has long been considered one of the most important traits for temperate adaptation [32]. Further GWASs for 27 phenotypes, including endosperm color, flowering time, and kernel development, identified several associated candidate genes with *cis*-regulatory elements in selection signals (Fig. 5). For all genes, either their *cis*-eQTLs, their *cis*-sQTLs, or both were within or close to the regions exhibiting strong selective sweep signals. For example, *y1* encodes a phytoene synthetase involved in carotenoid biosynthesis [58] (Fig. 5a, e). This gene is associated with endosperm color via GWASs (this study) and transcriptome wide association studies (TWAS) [81] (Fig. 5a). Temperate maize was specifically selected for the *y1* allele, resulting in the greater accumulation of carotenoids in the endosperm compared to that of tropical maize [82] (Fig. 5b). We showed here that *zmsweet2* is associated with flowering time, and the upstream eQTL is nearby a local XP-CLR peak, in addition to one sQTL in the gene body near another selective sweep signal peak (Fig. 5f). Its paralog *zmsweet4c* was shown to be involved in seed filling, while *zmsweet13s* was also shown to be associated with flowering time [83]. The results in this study suggest *zmsweet2* as a promising candidate gene for flowering time regulation. We identified *mcm4* (*Minichromosome maintenance protein 4*) (Zm00001d009374) as another candidate gene for flowering time (Fig. 5g). Consistent with the characterization of this gene’s Arabidopsis homolog, Zm00001d009374 is highly expression in flowering tissues such as pollinated internode, silks and female spikelet (Additional file 1: Fig. S10). In Arabidopsis, mutations in *BICELLULAR POLLEN1* (*BICE1*) lead to defective gametogenesis and it was shown to play a role in modulating DNA replication by interacting with MCM4 [84]. The cell cycle is coupled with cell fate specification. These observations suggest at least a potential connection between DNA replication and changes in flowering time. Zm00001d049559 encodes a transaldolase that we identified here as a candidate gene associated with kernel development with strong selection signals for expression and eQTLs (Fig. 5h). This gene is an ortholog of Arabidopsis Clc-HYPERSENSITIVE MUTANT2 (GSM2)-like [85]. Arabidopsis GSM2 localizes to cotyledon chloroplast and contributes to scavenging reactive oxygen species in response to glucose during cotyledon development [85]. Transaldolases act one step upstream of transketolase to catalyze the oxidation of glucose-6-phosphate into ribulose-5-phosphate, which is a critical process in the oxidative pentose phosphate pathway (PPP). Defective PPP in chloroplasts is associated with lower oil and starch contents in the embryo [86]. Together, these data provide insights into one potential mode of action for *cis*-regulatory elements involved in maize temperate adaptation.

In conclusion, this study systematically investigated maize global transcriptome gene expression heritability, which, advances our understanding of how variation in gene expression may have supported the adaptation of maize to temperate environments. Our deployment of the ICA method in this study showed promising results in exploring potential latent *trans*-regulatory modules in the maize genome to capture missing heritability. Even though the genome assembly and annotation used in this study (the B73 genome version 4) represent the gold standard of a maize genome, structural variation such as presence/absence variation (PAV), copy number variation (CNV) and insertion/deletion (Indels) can affect the number of reads mapped to each genomic region. Future work should be therefore focus on pan-genome eQTL mapping to capture more gene expression heritability in maize.

## Methods

### Plant growth conditions

Plant tissue for this study was collected from maize plants grown between May 2017 and October 2019 in overlapping batches of 16 genotypes. Kernels were surface sterilized with chlorine gas in a desiccator. Surface sterilized kernels were hydrated in aerated 1 mM CaCl_2_ solution overnight before transfer to petri dishes containing paper towels soaked with 1 mM CaCl_2_. Petri dishes were sealed with micropore tape (3 M) and wrapped in black cloth before being placed in an incubator at 28–30 $^{\circ }$C for 4–5 days to allow kernels to germinate.

Each sample was generated from two kernels of the same genotype that produced both radicle roots and coleoptiles (successful germination) and were transfered to a hydroponic growth system consisting of a glass tube filled with 3 mm diameter glass beads and encased in PVC pipes to maintain dark conditions for the seedling root system. All of the 16 PVCs pipes representing the 16 different maize lines were embedded in a rack platform connected to an intermittent watering system containing essential nutrients to support seedling growth (see Supplemental Table S3 for the recipe of the nutrient solution). The hydroponic growth system was placed in a growth chamber with 60% relative humidity, a 16-h-light/8-h-dark cycle, and 26$^{\circ }$:8$^{\circ }$ day and night target temperatures.

Root tissue was harvested from the seedlings at 14 days of growth in the hydroponic system, between zeitgeber time 5 (ZT5) and ZT8 (with ZT0 being subjective dawn), and flash frozen in liquid nitrogen before storage at − 80 $^{\circ }$C until RNA extraction. Root tissue collection was conducted in a dark room solely illuminated by a bulb covered by a green filter (Cinegel #4490, Grand Stage Company, Chicago, IL). Biological replicates consisted of different sets of two kernels of the same genotype grown as part of different batches at different times between the start and end of the 29-month data collection period.

### RNA extraction and sequencing

Frozen root samples were homogenized by grinding to a fine powder in liquid nitrogen. Total RNA was extracted with Trizol reagent from approximately 50 mg as per the manufacturer’s instructions. Total RNA was precipitated by centrifugation at 12,000 g for 15 min at 4$^{\circ }\varvec{C}$. The resulting pellet was washed three times with 75 % (v/v) ethanol before being resuspended in 40 $\upmu$L of DEPC−treated water heated to 65$^{\circ }\varvec{C}$.

RNA samples with RNA integrity number (RIN) < 5 (Agilent 2100 Bioanalyzer) were discarded and new extractions conducted. RNA-seq libraries were constructed using the Illumina TruSeq v2 kit following the manufacturer’s published protocol [87], pooled, and sequenced on an Illumina Nextseq 500 instrument with a target read length of 2x75 nucleotides and a target sequencing depth of 20 M paired-end reads per sample.

### Quantification of gene expression

The overall quality of RNA-seq reads was assessed by FastQC [88]. Reads assigned to each RNA-seq library were filtered and quality trimmed using Trimmomatic (v 0.33) with parameter settings “-phred33 LEADING:3 TRAILING:3 slidingwindow:4:15 MINLEN:36 ILLUMINACLIP:TruSeq3-PE.fa:2:30:10” [89]. Trimmed reads were mapped to the B73_RefGen_v4 maize reference genome [90, 91] using STAR (v2.7) in two rounds [92], as implemented in golden_map.py (https://github.com/yuhuihui2011/MEPsuite).

A combination of python scripts (deposited in the github repository associated with this article) and the “prepDE.py” python script provided by the StringTie (v2.1) [93] package were employed to generate read counts. Estimated fragments per kilobase of transcript per million mapped reads (FPKM) values for all libraries were exported by Ballgown (v2.20.0) [94].

### Genotype dataset preparation

A set of 12,191,984 markers with a minor allele frequency $\ge$ 0.05 and heterozygous genotypes call frequency $\le$ 0.02 was generated for use in this study via a combination of published markers from whole genome resequencing data, SNP calling from RNA-seq data, and imputation. Whole genome resequencing data was sourced from 622 lines genotyped as part of the maize HapMap3 project [44], including 110 lines for which RNA-seq data was generated as part of this study. The remaining 588 lines of the maize HapMap3 project were excluded based on either excessively levels of missing data ($\ge$ 0.6) or lower than expected inbreeding coefficients ($\le$ 0.9). A provisional marker set was generated by removing HapMap3 markers with an observed minor allele frequency < 0.01 among the remaining lines or a missing data rate > 0.6. These markers with imputed using Beagle/5.1 with parameter settings “window=1 overlap=0.1 ne=1200” [95] and further filtered to remove markers with a frequency of heterozygous calls > 20%. These SNPs were called across all samples used for RNA-seq using the alignments produced by STAR (described above) and GATK4 (v4.1) in GVCF mode [96]. Missing data for RNA-seq samples were imputed as described above. In cases where an individual RNA-seq sample yielded heterozygous calls for > 20% of genotyped markers the samples was discarded from all downstream analyses and considered to represent genetic (pollen), sample (RNA isolation), or barcoding (library construction) contamination. A final filtering step removed markers with a heterozygous genotype call frequency > 0.02 or a minor allele frequency < 0.05, resulting in the final set of 12,191,984 markers.

A kinship matrix was calculated by first identifying a set of 244,683 SNP markers in low LD with each other (*R*^2^ $\le$ 0.2) using PLINK (v1.9) [97]. Principal components of genetic variation were calculated using a randomly selected set of 1,000,000 markers from the total set of markers using tassel/5.2. LD heatmaps were generated using the R package Gaston [98].

### Gene expression heritability

Broad sense heritability for the expression of individual genes was estimated as the total variation explained by genotype (sigmal_G) as a proportion of total variation (sigma_P) ($H^2=\sigma _G^2/\sigma _P^2$) [68]. sigma_G and sigma_P were estimated using the lme4 R package and data for 219 replicated genotypes or separately for a set of 22 out of 40 (22 with replicates) genotypes of high confidence tropical origin and 39 out of 52 (39 with replicates) genotypes of high confidence temperate origin [42], fitting genotype as a random effect [99]. All other unexplained variations were considered as random errors.

To test for significant shifts in the heritability of genes in the same functional groups (same GO terms) in the whole RNA-seq population (340 genotypes), a two-sample Kolmogorov-Smirnov test was performed using the heritability of genes assigned the same GO annotations published as part of the Maize-GAMER dataset [100] and the heritability of other genes assigned to the other GO terms. For testing purposes, the population set was defined as 19,565 genes with FPKM $\ge$ 1 in more than 80% of all RNA-seq samples included in this study. The resulting *p* values for GO terms were corrected for FDR <0.05 (Benjamini-Hochberg method of multiple testing correction), and the median heritability of the genes assigned to the test GO terms with a $\ge$ 20% difference compared to the background population was considered significant. For the purpose of visualization, the redundancy of significant GO terms identified by this method was reduced by REVIGO with the default method (SimRel) and mall similarity (0.5) [101]. The original list of GO terms is provided in Supplemental Table S2.

For genes with expression heritabilities reduced over 80% in temperate maize compared to those in tropical maize, GO term enrichment among these genes was performed by goatools with the background set defined as the 19,565 genes described before [102]. *p* values for each GO terms were corrected for a FDR < 0.05 (Benjamini-Hochberg method of multiple testing correction), and GO term redundancy was reduced in REVIGO with the same settings as above [101] with further manual optimization for visualization. The original list of GO terms is provided in Supplemental Table S4.

### eQTL mapping and unique peak grouping

eQTL mapping was conducted using MatrixEQTL(v2.3) [43]. For each distinct genotype, average values across biological replicates were calculated. For each gene, expression values were transformed using the Box-Cox method [103] prior to mapping. Five principle components of population structure included as covariates. For each gene, eQTLs were classified as *cis* if they were located within 1 Mb upstream or downstream of the gene’s annotated transcription start site or transcription stop site. *p*-values for each gene-SNP pair were adjusted using Bonferroni correction (*p* = 4.1e^-9^) [104]. Individual SNPs associated with a given trait were grouped into peaks when sequential statistically significant SNPs occurred with a separation distance of < 1 Mb. When multiple SNPs were grouped into a single peak, the single most significantly associated SNP was labeled the “peak SNPs” and used as the representative of the entire peak in downstream analyses. eQTL peaks with at least 3 significantly SNPs were retained for downstream analyses.

### Permutation-based threshold for *trans*-eQTL identification

The suitability of the multiple-testing corrected statistical significance threshold employed for *trans*-eQTL was evaluated via permutation. Permutation testing was conducted by randomizing the labeling of the sample IDs in the genetic marker data file, while retaining the correct sample ID labeling of both the phenotype (e.g., e-trait) and population structure PC files. eQTL mapping was conducted using the permuted genetic marker file and non-permuted expression matrix as described above. For each gene/e-trait, the *p*-value of the single most significant SNP anywhere in the genome was retained. A permutation based *p*-value threshold for *trans*-eQTL was determined by identifying the 95 percentile of the distribution of negative logarithm base 10 transformed permutation *p* values.

### Quantification of RNA splicing and splicing-QTL mapping

Introns used in this analysis were assembled by StringTie (v2.1) [93] based on the reads mapped to the B73_RefGen_V4 reference genome using STAR [92] (as described above). For each putative intron, we calculated the percent-spliced-in (PSI) value, defined as the count of exon-exon junction reads normalized by the average per base read coverage of all reads in the intron. The formula used to calculate PSI was ucount/(depth/size), where ucount is the number of uniquely mapped junction reads for an intron using the iexpr function in Ballgown (v2.20.0) [94], depth is the sum of uniquely mapped read depths per base for the intron calculated using samtools bedcov function (-Q 255) [105], and size is the length of the intron in base pairs. Based on the reads mapping to intronic regions retrieved from the transcripts assembled by StringTie (v2.1) [93], we identified 88,888 variable splicing events supported by at least five junction reads in at least 5% of all RNA-seq samples with standard errors of PSI $\ge$ 0.01. PSI was set to missing if depth was zero. sQTLs were called using the same method proposed for eQTL mapping, significant hits with allele affects (beta) higher than 0.05 were kept for peak consolidation using the method described above.

### Analysis of selective sweep genes under regional domestication

The fixation index (Fst) values were calculated from the SNP dataset for the 40 maize genotypes with high-confident tropical origins and 52 maize genotypes with high-confidence temperate origins using VCFtools (v0.1.16) with a window size of 100 kb and a step size of 10 kb [106]. Using the same set of SNP, whole-genome cross-population composite likelihood ratio (XP-CLR) scores were calculated using xpclr (v1.0) [107] to compare tropical and temperate populations using parameters “-w1 0.0005 100 100 1 -p1 0.95” for each chromosome following previous studies with modifications [38, 108, 109]. The genetic position of each SNP was inferred from a published genetic map constructed for the nested association mapping (NAM) population of US inbred lines assuming uniform rate of recombination between mapped markers [110]. To enable comparisons between Fst and XP-CLR scores, XP-CLR scores were averaged within each of the 100 kb Fst window [108]. For both Fst and XP-CLR, the top 10% of 100 kb windows were considered highly differentiated. Following a previously proposed QC protocol, windows with ratios of nucleotide diversity ($\pi$) (tropical/temperate) lower than the genome wide average were removed from the sets of both Fst and XP-CLR highly differentiated regions [108, 109]. A gene was considered a candidate target of selection for temperate adaptation if it was located entirely within windows identified as highly differentiated by both Fst and XP-CLR analyses. The R package Gaston was used to visualize LD (linkage disequilibrium) heatmaps in the candidate gene regions [98].

### Organismal phenotype GWAS

A set of 27 trait datasets with data for *geq* 250 maize genotypes for which RNA-seq data was generated as part of this study were obtained from USDA GRIN (https://www.ars-grin.gov/) or Peiffer et al. [111]. Raw trait values were Log transformed prior to analyses. GWAS was conducted using the algorithm GEMMA (v0.98.3) with a kinship matrix and the first five principal components of population structure included as covariates [45]. Genes considered potentially causal were (1) those where either a trait associated SNP or a SNP in > 0.8 LD with a trait-associated SNP was located within 50 kb of the annotated start and stop position of the gene or (2) those where an eQTL identified for expression level of the gene was also identified as a trait associated SNP and in > 0.8 LD with a trait associated SNP.

### Co-expression gene modules identified by independent component analysis

Independent component analysis was conducted with the FastICA algorithm as implemented in the FastICA package (version 1.2-2) [112, 113]. 16,972 gene models with broad sense heritability higher than 0.05 with expression standard deviation among 572 samples higher than 1 were used for downstream analysis. Based on singular value decomposition (SVD), 166 components explained 80% of the total variance in the expression matrix of the 16,972 selected genes. ICA was conducted using parameter settings “nbComp” = 166, “maximum iteration” = 500 and the default function “logcosh”. A kmean cluster applied to coefficient assigned to each individual divided 572 individuals into two clusters for each ICs calculated, the ICs where both clusters have more than 10 individuals were kept for downstream analysis. Final filtering applied was the ICs with a kurtosis value > 6 resulting in 42 components for downstream analysis. The genes with a FDR lower than 0.001 were considered to belong to a cluster under each IC. Genes associated with the module at an FDR threshold of 0.01 were considered as members of the co-expression module associated with the IC. The coefficients of these 42 independent components assigned to each of the 572 individuals were used to calculate the best linear unbiased predictions (BLUPs) using the lme4 package [99] resulting in IC coefficients for 340 genotypes which were used phenotypes for GWAS. Significant signals were consolidated into distinct peaks using the same method as described above. Peaks supported by fewer than three significant SNPs were excluded from downstream analysis. Genome-wide eQTL mapping was conducted using the SNPs in the peak using a relaxed significant threshold (10e−5) followed by fisher exact test of four categories: e-traits included in the IC, e-traits not included in the IC, genes in IC but not e-traits and gene not included in the IC but not e-traits. Fisher exact test *p* values represent e-trait enrichment level for the peak identified by ICA GWAS (see the “Methods”). Significant *p* values ($\le$ 0.01) indicate significant tendency of regulating the genes included in the corresponding independent component by the GWAS peak. Candidate causal genes were searched within 1 Mb flanking the lead SNP of each distinct peak of interest and were prioritized by the proximity to the lead SNP or the SNPs highly linked with the lead SNPs(LD R_2_ *geq* 0.8) and their characterized function. Gene Ontology (GO) enrichment analysis was performed using goatools [102] with 19,565 genes as population background. The resulted significant GO terms were then curated to reduce the redundancy using revigo (http://revigo.irb.hr/) [101] and manual examination.

## Supplementary Information

**Additional file 1:** **Supplementary Figures 1-11.**

**Additional file 2:** **Supplementary Table S1.** Recipe for making the nutrient solution to support plant growth in this study.

**Additional file 3:** **Supplementary Table S2.** Go terms showing significant shifts in broad sense expression heritability shown in Fig. 1.

**Additional file 4:** **Supplementary Table S3.** Classification of temperate or tropical panels among the genotypes used in this study.

**Additional file 5:** **Supplementary Table S4.** GO terms over-represented in genes showing declined expression heritability in temperate panels.

**Additional file 6:** **Supplementary Table S5.** Genomic regions with significant selective sweep signals identified by XP-CLR and Fst analysis.

**Additional file 7:** **Supplementary Table S6.** Phenotypic data collected for the genotypes included in this study.

**Additional file 8:** **Supplementary Table S7.**

**Additional file 9.** Peer review history.

## Data Availability

RNA-seq data for root tissues of 572 maize genotypes are available at NCBI under the BioProject: PRJNA793045 [114]. In-house code for major analysis and figure generation are accessible at Github: https://github.com/xiaoguanghuan123/maize_eGWAS and Zenodo: https://zenodo.org/record/7653569 [115]. The SNP dataset generated in this study can be retrieved from figshare: https://doi.org/10.6084/m9.figshare.19126139.v1 [116].
