# Supplementary material for: A role for heritable transcriptomic variation in maize adaptation to temperate environments
Source: Genome Biol. 2023 Mar 24;24:55. doi: 10.1186/s13059-023-02891-3 (PMC10037803; doi:10.1186/s13059-023-02891-3)
Supplement: Supplementary file 1 — Additional file 1: Supplementary Figures 1-11. [file 13059_2023_2891_MOESM1_ESM.docx]

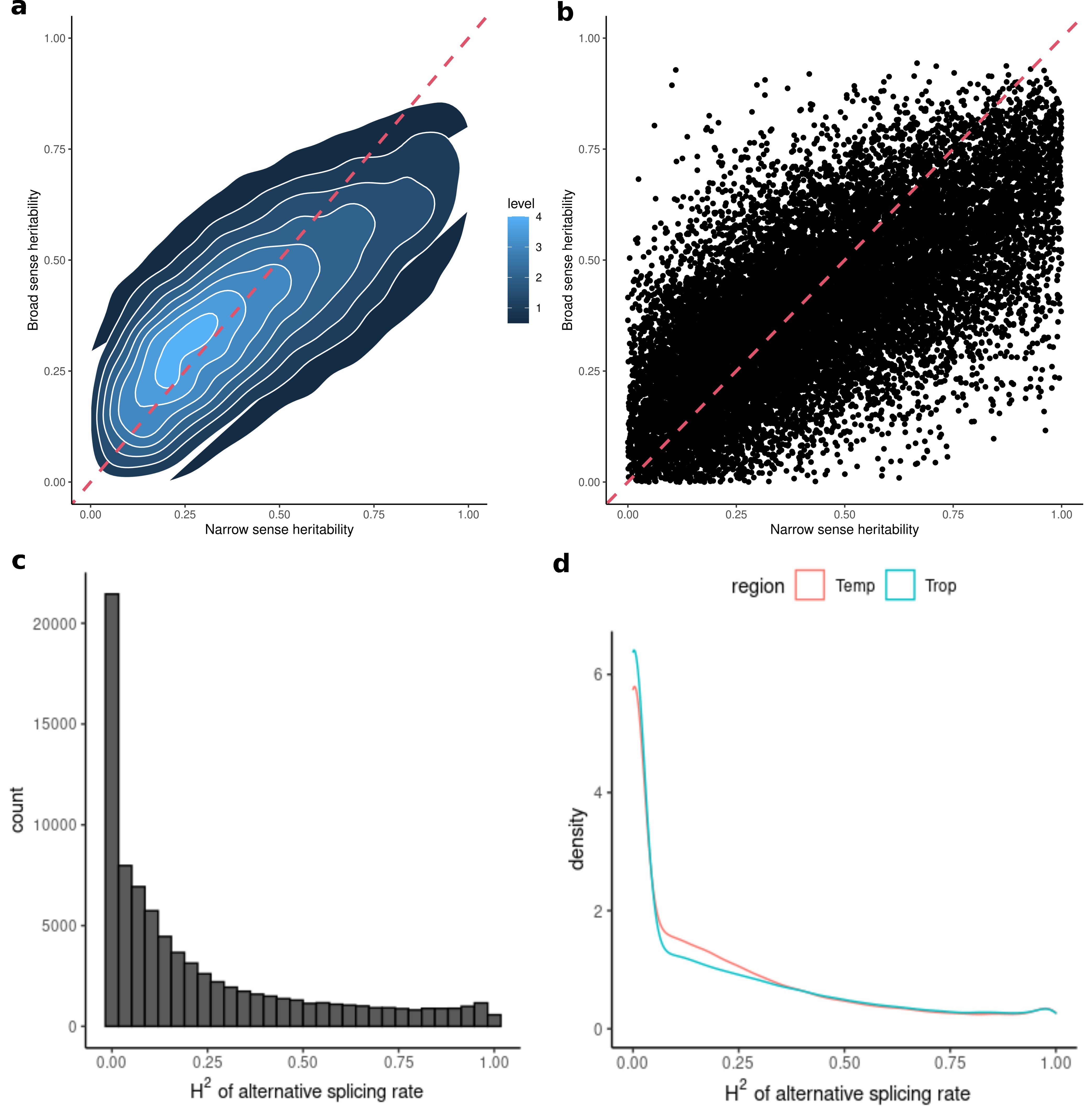


**Figure S1.** The heritability of gene expression and alternative splicing in maize lines (a-b) Correlation between broad sense heritability – individual sample level expression – and narrow sense heritability – for average expression level across all replicates of a given genotype – calculated for the 19,565 expressed genes in this study. Note that because here h^2^ is based on the mean expression per genotype and H^2^ based on the variance of expression across individual samples h^2^ it is theoretically possible for estimated h^2^ to exceed H^2^. Red dashed line indicates the x=y relationship corresponding to H^2^=h^2^. (c) Distribution of broad sense heritability of alternative splicing based on PSI values of 82,028 introns. (d) Distribution of broad sense heritability of alternative splicing based on PSI values of 82,028 introns in temperate and tropical maize.


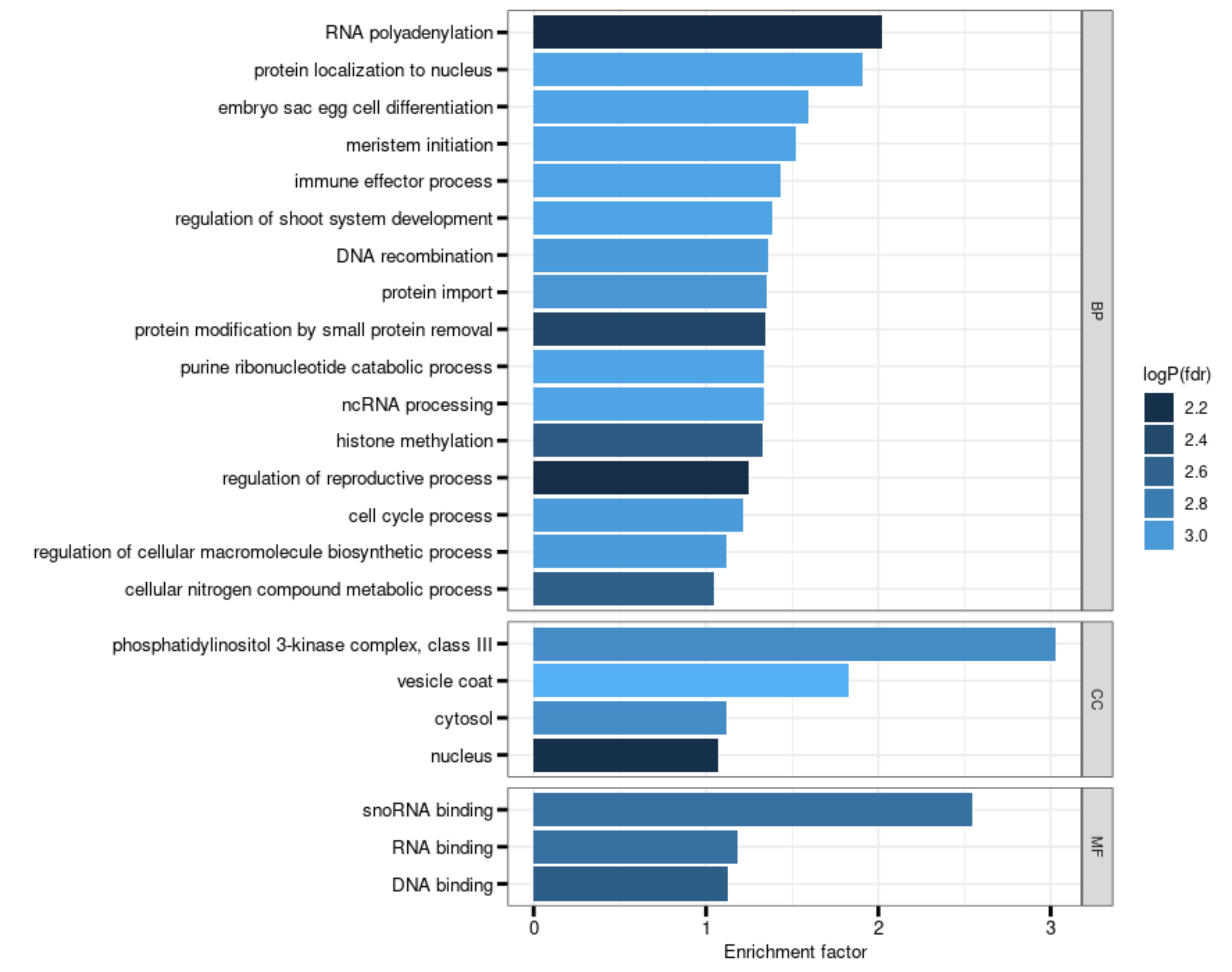


**Figure S2.** Gene Ontology terms enriched in the genes exhibiting reduced expression heritability in temperate maize. Significantly enriched GO terms (FDR ≤ 0.05) for 1,272 genes exhibiting at least an 80% reduction of expression heritability in the temperate maize population relative to the tropical maize population. Bars indicate the enrichment factor (number of genes associated with the overrepresented GO term in the study gene set over the number of genes associated with the GO term in the background gene set). The background gene set is the 19,565 genes used for the eGWAS. Negative Log_10_-transformed multi-test corrected *p*-values are color-coded.


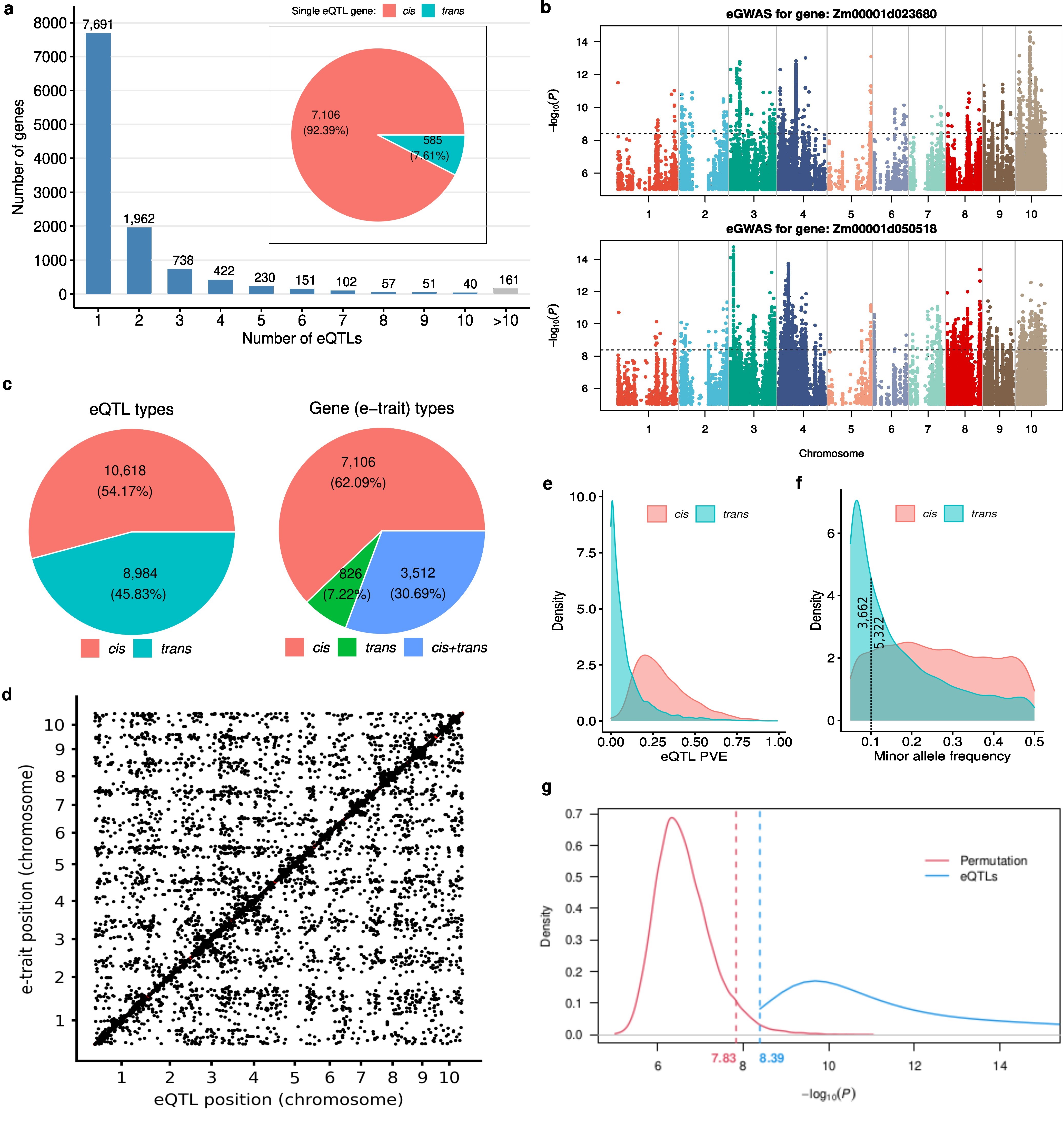


**Figure S3.** Global pattern of eQTL and false positive control (a) Number of genes with one or more significant peaks and the proportion of *cis*- and detected *trans*-eQTLs in genes with only one peak (pie plot). (b) Manhattan plots of eGWAS for two example genes with more than 10 peaks. (c) Distribution of minor allele frequency of *cis*-eQTLs and *trans*-eQTLs. 3,662 *trans*-eQTLs are with minor allele frequencies ≤ 0.1 and 5,322 *trans*-eQTLs have minor allele frequencies *>*0.1. (d) Distribution of expression variation explained by *cis*-eQTLs and *trans*-eQTLs. (e) The proportion of *cis*- and *trans*-eQTLs identified by eQTL mapping in this study. (f) The proportion of e-traits with single *cis*-eQTL, single *trans*-eQTL and both. (g) P-values of the association between the SNP dataset of which the genotype were permutated and gene expression calculated by matrixEQTL and the overlap between the p values of eQTLs identified using the original dataset in this study. 95 percentile of the minimum p-values (-log10 transformed) of random permutation of all 19,565 genes is corresponding to 7.83 and the p-value cutoff used for eQTL mapping in this study is corresponding to 8.39 (-log10(0.05/12191984)).


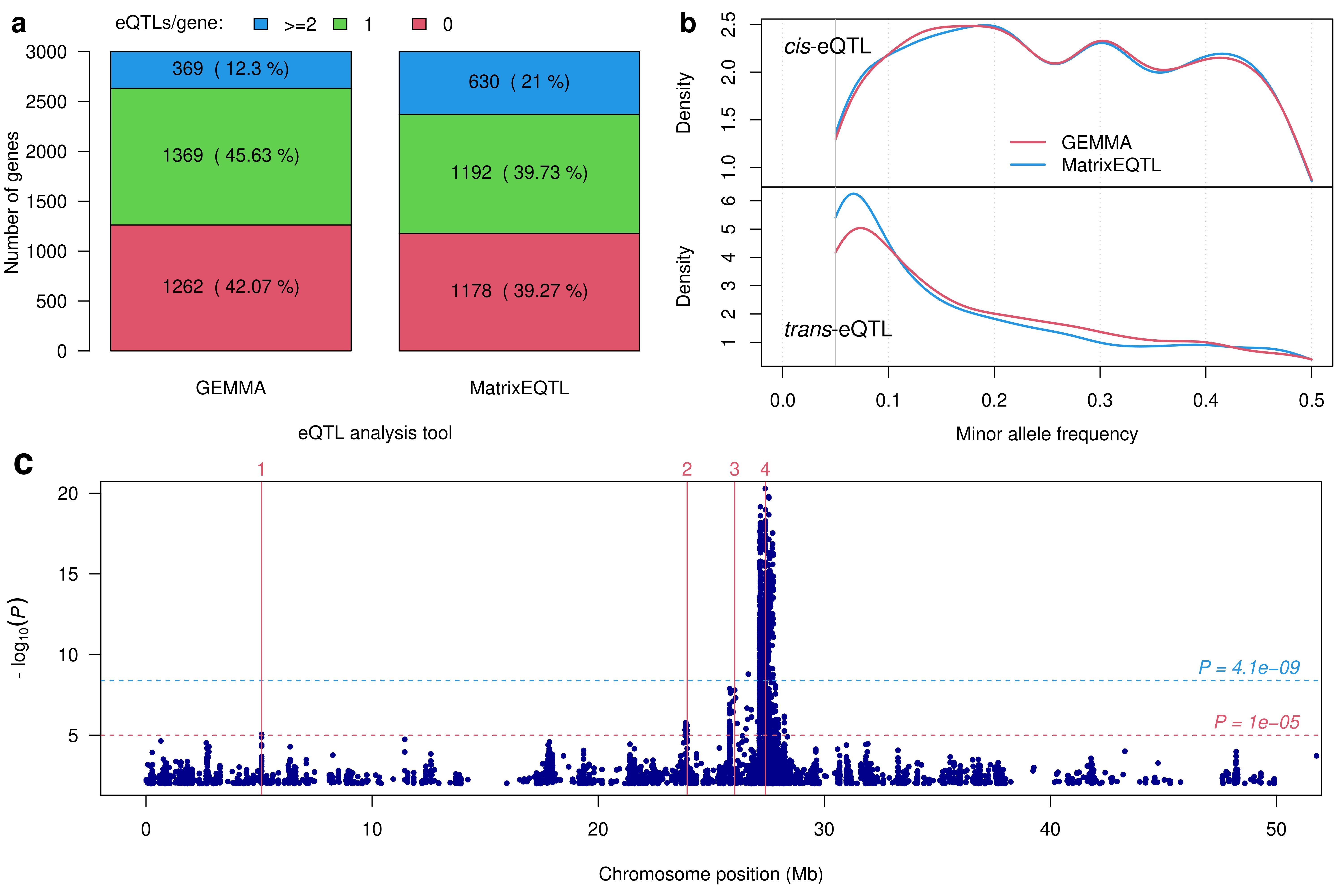


**Figure S4.** Comparison of eQTLs identified by GEMMA and matrixEQTL for a subset of 3,000 genes (a) Number of genes with 0, 1 and more than 1 eQTLs identified by GEMMA and MatrixEQTL. (b) Minor allele frequency of *cis*-eQTL and *trans*-eQTL identified by GEMMA and MatrixEQTL. (c) Different number of peaks identified by GEMMA and MatrixEQTL. Manhattan plots is generated based on the p-values from Mixed Linear Model (MLM) implemented in GEMMA for gene Zm00001d000501. GEMMA identified only one eQTL on chromosome 3 at the threshold 4.10e-9 (blue dashed horizontal line), while MatrixEQTL identified four (red vertical lines). However, there are weak peak signals above 1e-5 (red dashed horizontal line), at the three eQTLs not identified by GEMMA.


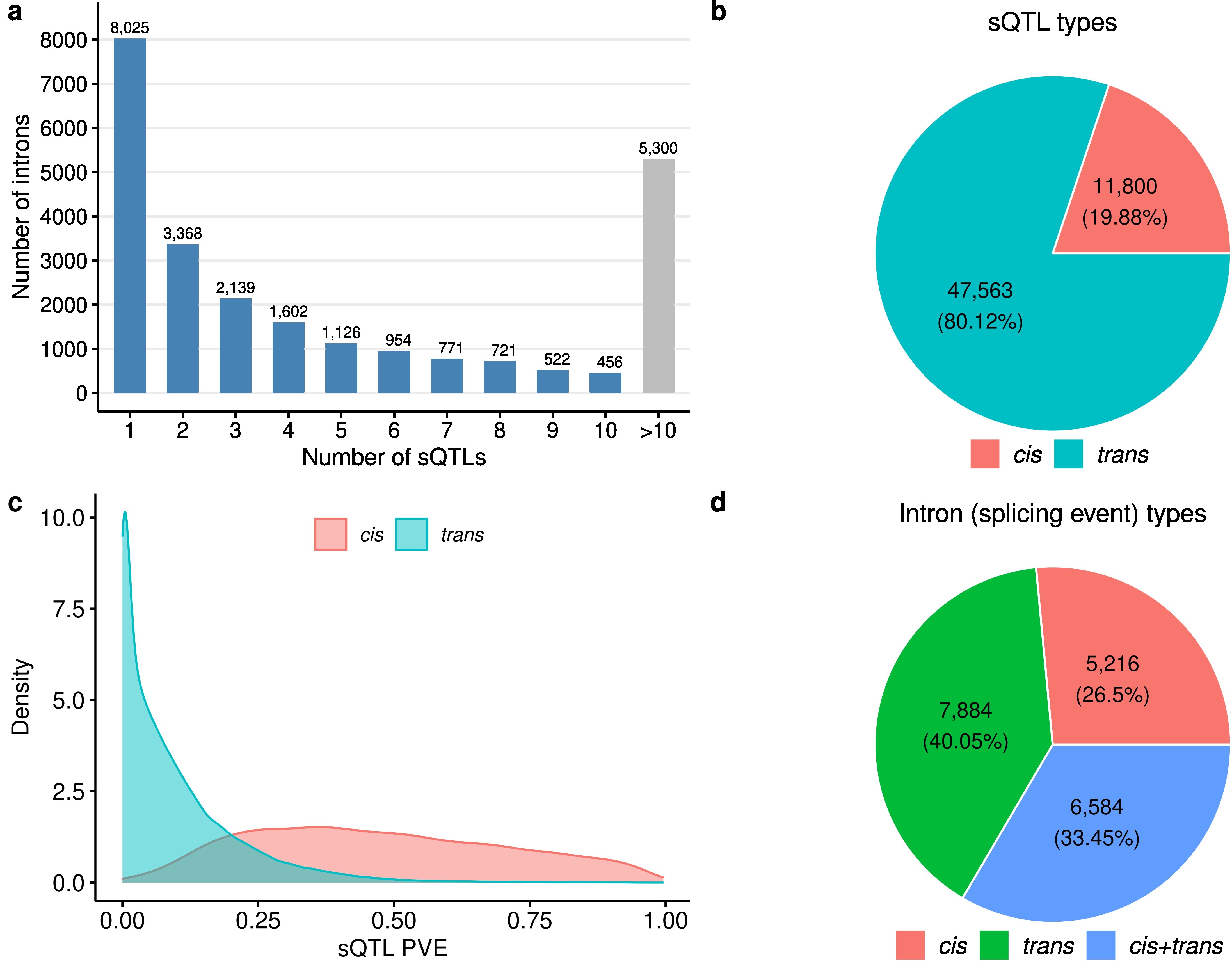


**Figure S5.** sQTL composition and distribution (a) Number of introns with one or more significant sQTL peaks. (b)

Expression variation explained by their associated *cis*- and *trans*-sQTLs. (c) Proportion of *cis*- and *trans*-sQTLs identified by sQTL mapping in this study. (d) Proportions among the 19,684 splicing events (introns) associated with just *cis*-, just *trans*-, or both *cis*- and *trans*-sQTLs.


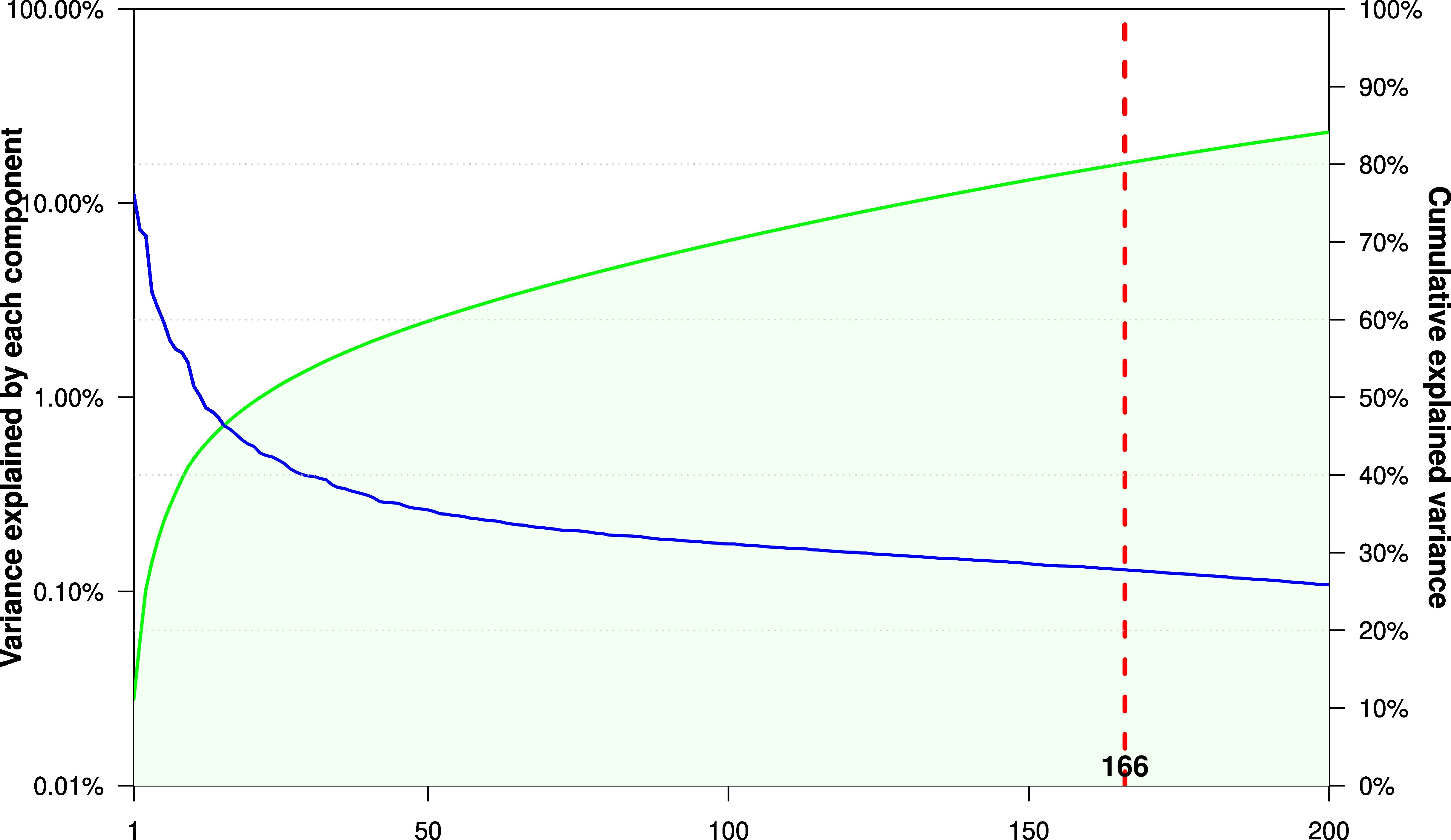


**Figure S6.** Percentage (blue curve) and cumulative percentage (green curve) of variance explained by independent components. The red dashed vertical line indicates the threshold 80% which can be explained by the first 166 components.


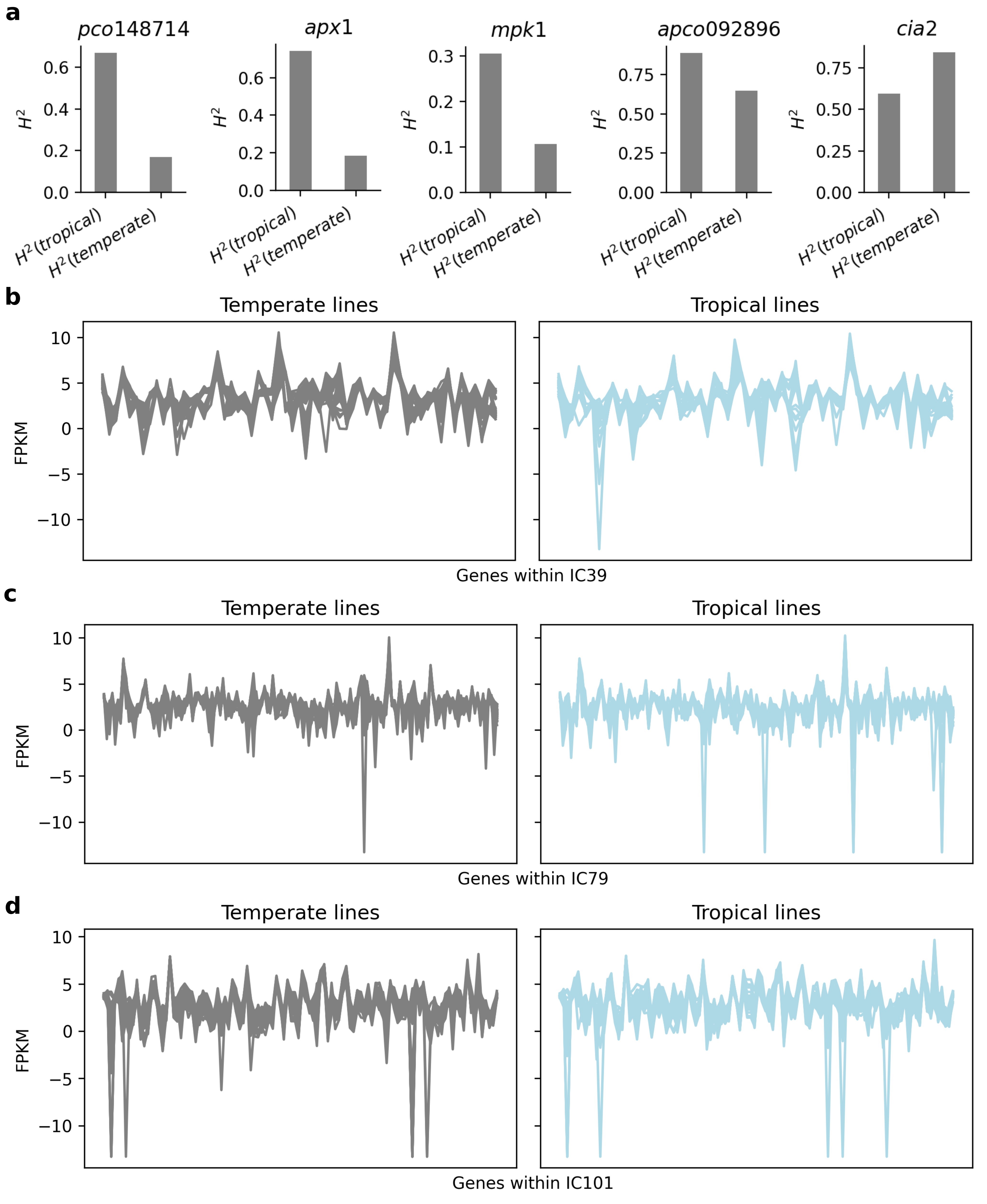


**Figure S7.** Expression patterns of the genes within each of the co-expression modules associated with IC39, IC79 and IC101. (a) Differences in expression broad sense heritability of five candidate genes shown in Fig. 3 in tropical and temperate maize. (b) Expression pattern of co-expression module associated with IC39 in temperate (left) and tropical maize (right). Each grey line indicates the log2 transformed expression pattern of a single gene significantly associated with IC39 across all temperate maize samples included in our study population. Each blue line indicates the log2 transformed expression pattern of a single gene significantly associated with IC39 across all tropical maize samples included in our study population. (c) Expression pattern of co-expression module associated with IC79 in temperate (left) and tropical maize (right), visualized as described in panel (b). (d) Expression pattern of co-expression module associated with IC101 in temperate (left) and tropical maize (right), visualized as described in panel (b).


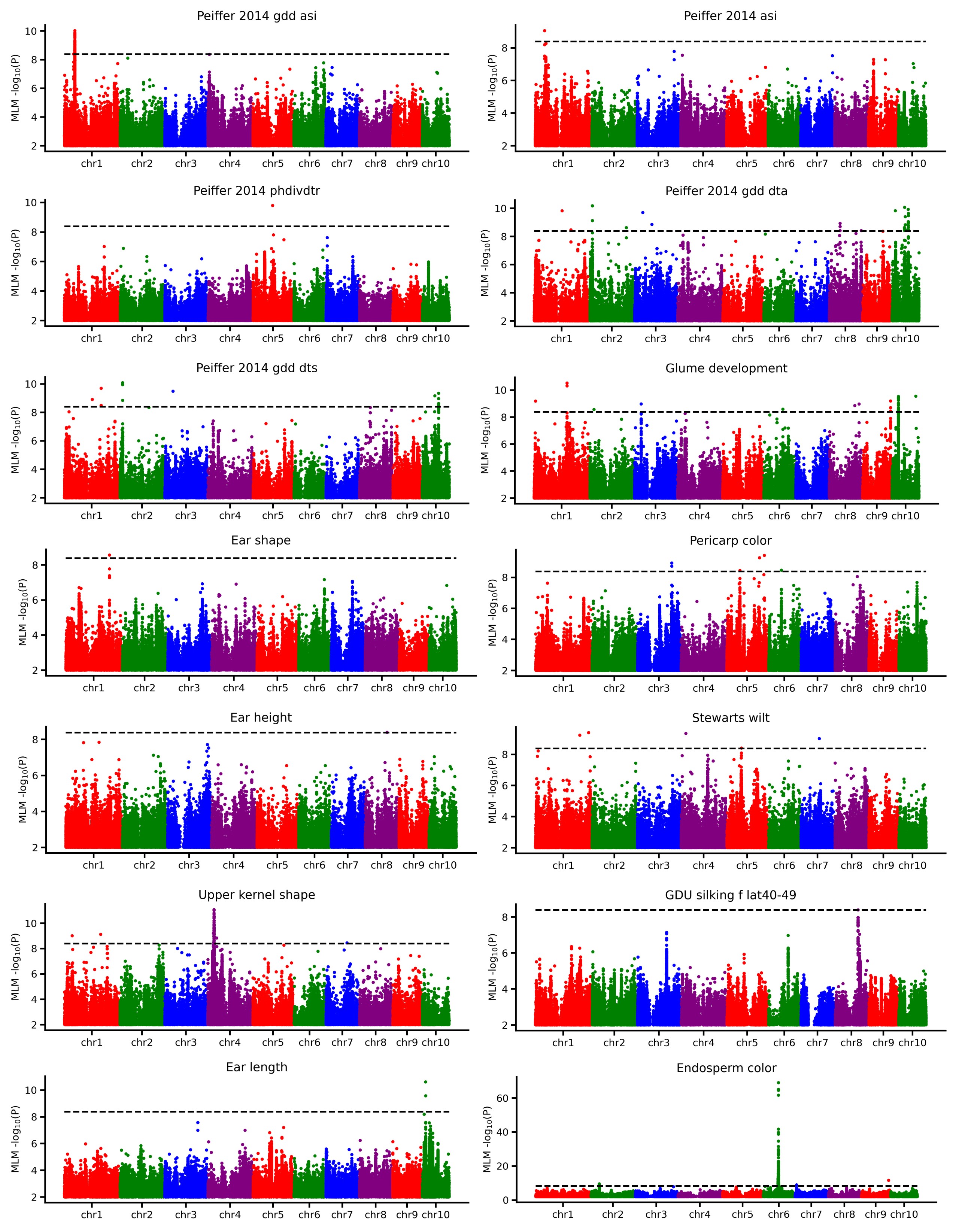


**Figure S8.** GWAS results of 14 organismal phenotypes used for this study.


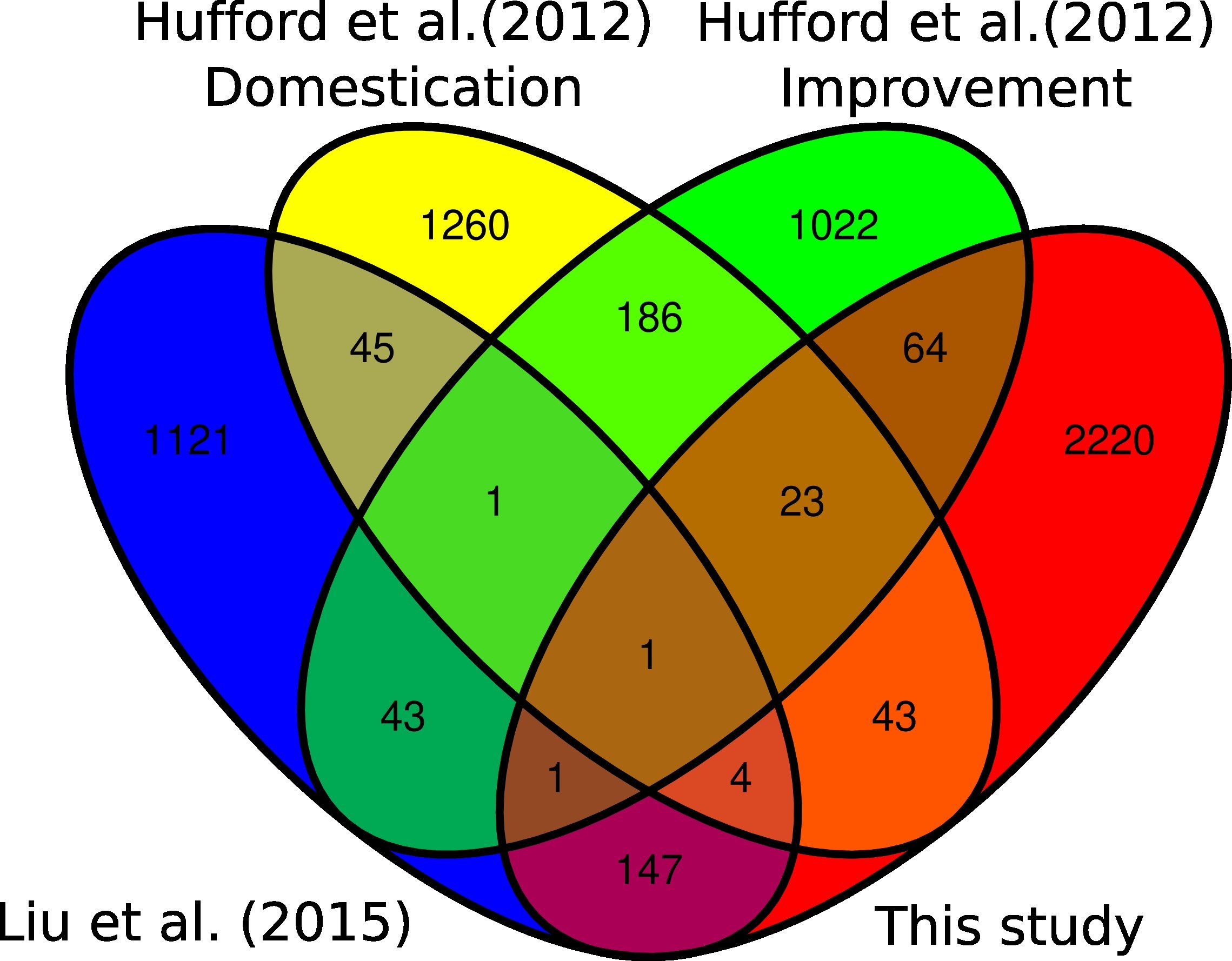


**Figure S9.** Gene sets identified by genome-wide selection analysis for maize domestication and improvement (Hufford et al. (2012)) and temperate adaptation (Liu et al. (2015)) and temperate adaptation conducted in this study. Gene IDs

were translated into B73 RefGene v4 based on the ID conversion in MaizeGDB database (https //www.maizegdb.org/gene center/gene#translate).


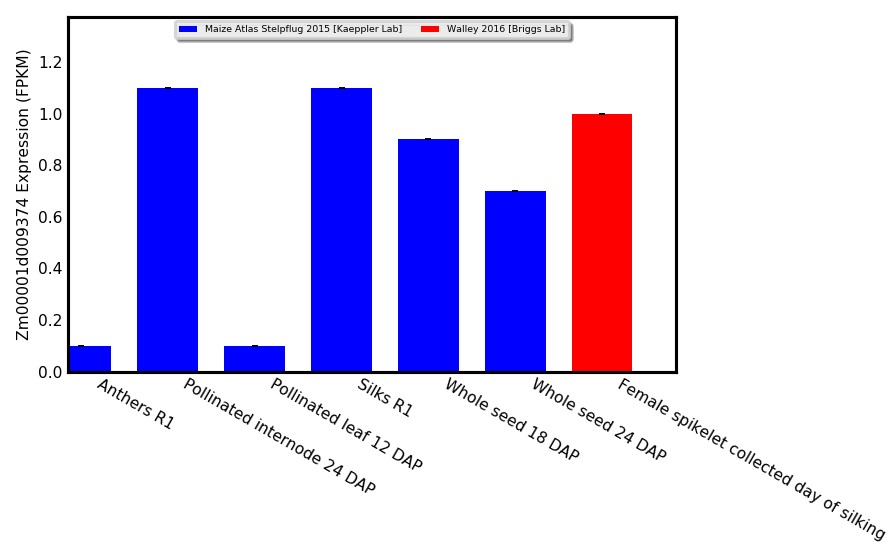


**Figure S10.** Expression patterns (FPKM) of *mcm4* across different flowering tissues in maize reference inbred B73


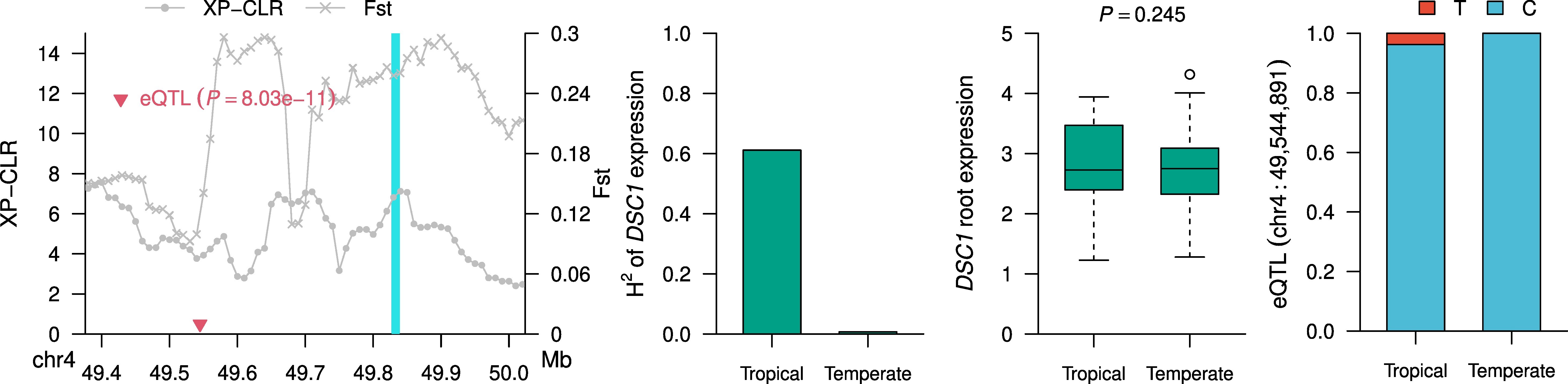
**Figure S11.** Selection features of *DSC1* associated with kernel development From left to right, selective sweep signals (XP-CLR and Fst), expression broad sense heritability (H^2^), expression level (FPKM) in roots, and *cis*-eQTL lead SNP allele frequency comparison between tropical and temperate maize
