# Supplementary material for: A role for heritable transcriptomic variation in maize adaptation to temperate environments
Source: Genome Biol. 2023 Mar 24;24:55. doi: 10.1186/s13059-023-02891-3 (PMC10037803; doi:10.1186/s13059-023-02891-3)
Supplement: Supplementary file 9 — Additional file 9. Peer review history. [file 13059_2023_2891_MOESM9_ESM.docx]

**Review history**

**First round of review**

**Reviewer 1**

For many decades the focus of studies of domesticated plants have been on the role of genes on phenotypic variation, this manuscript starts to bridge the gap between genes and phenotypic data in maize. The design includes the best studied and longer adaptation to temperate environments and used two groups of genetic lines, "temperate" and "tropical". The roots from 340 lines or genotypes were explored for expression heritabilities, eGWAS and sGWAS. Results from 572 root samples show variation in broad sense heritabilities for different sets of genes from 259 samples which are replicas from two or more times. While results are complex and have several angles, the reading of the manuscript was pleasant and permits the reader to begin to understand the architectural organization of the expression of maize genes in particular the importance of transcriptional regulatory networks in temperate adaptation. I have some comments which would improve the presentation of the results and their discussion.

Specific comments
Page 4, line 54, substitute "P ¡" with "P <"
Page 5, line 20, substitute "P ¡" with "P <"
Page 5, lines 27-29, this is confusing, please provide the evidence for population structure and "other confounding factors"
Page 7, lines 24-25, not clear why 0.33 is modest, it is still in disequilibrium, probably you can delete "modestly linked" and substitute it with "in disequilibrium"
Page 7, line 60, substitute "WE" with "We"
Page 12, line 11, substitute "Parviglumis" with "parviglumis"
Page 13, line 33, substitute "temperat" with "temperate"
Table S1, sometimes you use P for statistical significance (in the text) but here you use p, like in this Table. I suggest that you use pin the whole manuscript

**Reviewer 2**

The authors identified the variations in maize gene expression and alternative splicing, by generating 572 RNA-seq datasets from the roots of 340 maize individuals. They identified numbers of eQTLs and sQTLs based on association analyses, and estimated the differences in the broad-sense heritability between the tropical and temperate maize groups. The whole project is well designed to include data repeats for reliable downstream analyses. The generated population scale RNA-seq data are good resources for the community. However, the manuscript was not well organized, the logic in the "Results" and "Discussions" sections are confused, which make the main points and highlights unclear. Please find below in more detail some major concerns as well as several minor issues.

Major

1. The whole manuscript is not well organized. The "Results" section is a mixture of "how we did these analyses" and descriptions of the basic distribution of the results, which from my point of view, the former part should be moved to the "Methods" section, while the later part should be curtailed and more biological-meaningful results should be added to highlight the main findings. Meanwhile, the "Discussion" section contains lots of conclusions that should be provided in the "Results" section.
For example, in Line 11-24 (Page 6 of 27), the authors spend lots of efforts to explain how the alternative splicing (AS) analyses were performed. To my knowledge, the AS were identified based on canonical PSI value based method with no special modifies, thus is needless to emphasis in the Results section.
In the "Expression quantitative trait loci reveal the genetic architecture of transcriptional regulation" sub-section (Page 5 - Page 6). The authors spend lots of works describing the results of eQTLs. There are mainly three results: i) identified 7,691 eQTLs including 7,105 cis-eQTLs and 586 trans-eQTLs; ii) there are no trans-eQTL hotspots and iii) trans-eQTLs represent more rare alleles. However, there lack discussions on the importance of these findings and the possible reasons that causing these results (biological or statistical? Based on the biased proportions of cis and trans eQTLs, it is reasonable to doubt that the association between rare alleles and trans-eQTLs may be caused by statistical bias, say if rare alleles are more like to occur in regions distant to genes. Thus, I suggested the authors to perform some enrichment analyses to support their findings). Besides, I did not see the connections between these findings and the other sub-sections of the results, as well as the main point (as inferred from the title) of "a role for heritable transcriptomic variation in maize adaptation to temperate environments".
I suggested the authors rearrange the Results and Discussion sections to simplify the descriptive statements, highlight the main points and how did them support the main findings of the study.

2. The authors identified genomics regions exhibiting significant selective sweep signals for adaptation to temperate environment, and listed several known gene cases that located in the select regions. However, the results lack the general genomic patterns of these select regions. The evidences of several gene cases are insufficient to support the sub-title (line 38 page 8 of 27) of "Genomic mechanisms underlying temperate adaptation". To my knowledge, the selection signals for maize adaptation to temp environment have already been studied by Hufford et al. (https://doi.org/10.1038/ng.2309) and Liu et al (https://doi.org/10.1016/j.molp.2015.01.016), while the later used population level RNA-seq from kernels of a maize association mapping panel (AMP) including tropical and temperate individuals, similar to the population used in the current study. Besides. there are also public data of the RNA-seq from leaf in response to drought resistance (https://doi.org/10.1186/s13059-020-02069-1), population level metabolomics (https://doi.org/10.1038/ncomms4438) and DNA methylations (https://doi.org/10.1186/s13059-019-1859-0) for the AMP population. Leveraging these multi-omics resources could help the authors draw more comprehensive conclusion on the temperate adaptation of maize. Why these resources were not considered in the authors' analyses? What are the new findings that the authors could bring to the readers with the new RNA-seq data from root than those from kernels and leaves in AMP population?

3. The authors discussed the differences between broad sense heritability and narrow sense heritability in the Discussion section (Line 42-49 Page 10 of 27). However, only the broad sense heritability was estimated in the authors results. The authors could also perform analyses on the narrow sense heritability differences between tropical and temperate maize, to draw more solid conclusions.

4. In Line 11-27 (Page 7 of 27), the authors illustrated two cases for the colocalization of eQTLs and sQTLs (also showed in figure 3e and f). I suggest the authors to dig deeper into the detailed causations of these colocalizations. Is the leading variant the causal variant? Does the expression and splicing variations caused by the same causal variation? Or at least, the authors could provide additional information in figure 3 to illustrate how did the expression and splicing alter along with different alleles.

5. The authors estimated the H2 for gene expressions and perform ICA for AS. What is the H2 for AS between tropical and temperate maize? Can ICA and ICs-based GWAS be applied to gene expressions?

Minor

1. There are several typos that should be avoided, to list a few
Line 44 (page 5 of 27): "... was typically lower the estimate of total variance explained ...";
Line 61 (page 7 of 27): "WE";
Line 51 (Page 8 of 27): "exhibitED";
Line 8 (Page 12 of 27): "showeing";

2. All the figure legends were not well organized. The basic representations of each sub-figure or track should be added, rather than directly using a conclusion of what the figure meant to the readers as the sub-title. For example,
Figure 1 "(d) Distribution of broad sense heritability of genes in the tropical and temperate panels;", what's the meaning of the red dash line?
Figure 2 "(e) Rare trans-eQTLs are associated with extremes in gene expression, as identified in the entire population.", this is more of a conclusion than a figure legend.
Figure 5 is not cited in the article, and some panels in Figure 6 are not cited.
I suggested the authors to review and re-organize all the legends to make them more informative.

3. Line 6-8 (Page 6 of 27): "These results are consistent with disregulation of expression induced by trans sites imposing similar fitness burdens as those previously observed at cis-regulatory sites." Please add references that support this conclusion.

4. Figure 6a contains too many points to be provided as vectogram. My pdf viewer spends extra time loading the paths of these points each time I navigated to this figure. Please reduce the paths in the vectogram or make it a bitmap image.

5. Line 41-42 (Page 7 of 27): what is the total variance explained by the retained 43 ICs?

**Reviewer 3**

The authors present and analysis of a large and high quality transcriptome dataset from diverse maize genotypes. The analysis focuses on identifying QTLs for expression and splicing, partitioning those between cis and trans, while studying whether the genetic architecture of expression has changed during the evolution of temperate lines from tropical lines. They show evidence of changes in the heritability of certain classes of genes, identify several interesting candidates with potential contributions to temperate adaptation, study the co-localization of expression and splicing QTL, and study the hypothesis of rare allele burden on expression.

Overall, I think the dataset is strong, and the analysis is well done. I particularly like the ICA analysis and think it's a power approach to these data. I do have some trouble following the main story of the paper - is it a study of temperate adaptation or a study of the architecture of expression variation? If it is both, I think the link between them can be made more explicit. I also have a major concern about the trans-eQTL results which I'll discuss below, and several questions and more minor comments on the presentation.

My major concern with the results is that I'm not convinced the trans-eQTL results are robust - they are likely dominated by false positives. My evidence for this is:
1. Figure 2c shows that the detected trans-eQTL explain very little variation. With a sample size of only 340 lines, the power to detect a QTL that explains 5% of the variation with a threshold of 4.1e-9 is only about 4%. This would mean that there should be 25x as many trans-eQTLs of similar magnitude to your discoveries in this population, which would be a very large number
2. Figure 2d shows that these detected trans-eQTLs explain little variation because the markers are very rare (modal maf < 0.1). This means that most of these trans-eQTLs are represented by only a few individuals in the dataset. It looks like you used a maf cutoff of 0.01 for the eQTL, meaning that you'd be testing markers with only 4 individuals represented by the minor allele. The problem with this is that the linear models get very sensitive to departures from normality of residuals when one of the alleles is rare (we lose the guarantees of the central limit theorem). The box-cox transform helps, but isn't flexible enough to ensure that residuals will be normal. You could try more extreme normalizations like rank-transformation or quantile-normalization. Genotype permutations could be used to test whether rare alleles are likely to give false positives - stratify the permutations by allele frequency and look at the false positive rate separately for maf < 0.05 and > 0.05; care is needed to ensure the permutations don't break population structure-phenotype associations. But the safest would be to simply throw out markers with maf < 0.05 (or maybe 0.1), perhaps guided by genotype permutations. Also, note that MatrixEQTL doesn't fit a mixed model, unlike the more complete GEMMA model used for other traits, and 5 PCs are less likely to pick up subtle relatedness that may generate correlations among individuals at rare haplotypes. You might try seeing if some of these rare trans-eQTLs remain significant if you fit them with GEMMA or some other full LMM.
3. Figure 2c/d shows that in contrast cis-eQTLs are not found with low PVE or low MAF. If you could accurately detect trans-eQTLs at maf<0.05, or pve < 0.05, why couldn't you similarly detect cis-eQTLs of the same effect sizes? The simplest explanation is that the proportion of cis-SNPs that have large effects is much greater (orders of magnitude greater!) than the number of trans-SNPs that have large effects. For cis-SNPs, if you have a false positive in 1/1e7 SNPs, but true positives in 1/1e4 SNPs, then your positives are dominated by true positives. But the number of SNPs-gene combinations that is a true trans-eQTL is probably much lower (say 1/1e8). In this case, your positives are dominated by false positives. One potential solution could be to calibrate significance thresholds separately for trans- and cis- eQTLs, or to use false discovery rates to select hits separately for trans- and cis- eQTL
4. Figure 2e shows that for trans-eQTL pairs, the number of rare alleles correlates with expression rank. This is similar to the analysis in the Kremling paper, but subtly different in important ways. Here, you're only including trans-eQTL pairs that you detect as significant. Figure 2d shows that for the vast majority of these the maf is in the "rare" category. The fact that this is an eQTL means that individuals that carry the rare allele have either higher or lower expression than individuals that carry the common allele. And individuals that carry the rare allele probably share a rare haplotypes that also has other rare alleles as well. So this is really a circular argument. The Kremling analysis was not circular because all cis-eQTL pairs were included whether or not they were "significant" in association analyses. That's impossible to do here because you would have to compare the ranked expression to rare allele counts across all windows of the genome which wouldn't be informative. Also, why was a 0.2 threshold used for "rare"? That's not very rare. Kremling used 0.05. I'll note that Figure 2f is hopeful in that it's hard to see why the smile would replicate in a different sample if it was driven by false associations. But it is possible that shared population structure between the two data partitions could lead to this (eg. Josephs et al https://doi.org/10.1534/genetics.118.301786)
In summary, I think more is needed to claim that the trans-eQTLs, and the rare allele burden results are more than just noise.

On the question of paper focus, I think the idea of looking for selective sweeps at the genomic and expression level is interesting, but could be developed further. I think the hypothesis here should be made more explicit, especially in the introduction. Lines 33-36 on page 10 describe one reason for a change in heritability. But there are other explanations including changes in genetic architecture due to drift or selection. If the frequency of functional variants decreases, Vg will decrease, decreasing h2 even if the environmental responsiveness is constant.

More minor comments and concerns:
- GWAS methods:
- how were replicates dealt with in eQTL analysis?
- it looks like in ICA replicates were included as samples, and then collapsed to BLUPs afterwards
- what about for individual gene expression traits? It looks like 2 seeds were grown per tube. Were these the two replicates, or were 2 tubes grown per line? If the former, these 2 seeds cannot be used to calculate broad-sense heritability because they share more than just genotype, also environment.
- Fig 2a - The PVE of an eQTL is likely to be over-estimated unless you re-estimate the effect size in a new dataset. This is worth mentioning and can explain why some values are above the diagonal
- Are the ICA SNPs in genes loading on the ICA axes? p8.top - by snps being "linked" to genes, I think you mean the snp is physically located in or nearby this gene, not as an eQTL, right?
- Fig 7: what is the vertical blue bar in the left-hand panels?
- Fig S5 - what is the blue vs grey lines? Are you showing ICA loadings for all genes here or only ones significantly associated with an IC?
- Figure 1d-e and 4.57. To ask whether H2 was lower in temperate vs tropical maize lines, you should use a paired t-test, or equivalently do a one-sample t-test on the difference between H2 values per gene. The regression slope doesn't give this. The R2 of the regression would be equivalent to the correlation between H2 values which could be interesting as well. Also, 1e is sort of a trivial result - of course the blue density is shifted left than the red density - genes with higher values of H2-tropical will tend to have values of H2-temperate lower than H2-tropical almost by definition. The only interesting addition to 1d I could imagine would be to show the densities of H2-temperate and H2-tropical along the margins of 1d.

**Authors’ response to reviewers**

Reviewers Text in Black

Author Responses in Blue

Reviewer #1: For many decades the focus of studies of domesticated plants have been on the role of genes on phenotypic variation, this manuscript starts to bridge the gap between genes and phenotypic data in maize. The design includes the best studied and longer adaptation to temperate environments and used two groups of genetic lines, "temperate" and "tropical". The roots from 340 lines or genotypes were explored for expression heritabilities, eGWAS and sGWAS. Results from 572 root samples show **variation** in broad sense heritabilities for different sets of genes from 259 samples which are replicas from two or more times. While results are complex and have several angles, the reading of the manuscript was pleasant and permits the reader to begin to understand the architectural organization of the expression of maize genes in particular the importance of transcriptional regulatory networks in temperate adaptation. I have some comments which would improve the presentation of the results and their discussion.

Thank you for the time you invested in reviewing our manuscript. We appreciate your feedback and the supportive comments about our work.

Specific comments

Page 4, line 54, substitute "P ¡" with "P <"

Corrected

Page 5, line 20, substitute "P ¡" with "P <"

Corrected

Page 5, lines 27-29, this is confusing, please provide the evidence for population structure and "other confounding factors"

We apologize for the confusion. This sentence skipped over a key intermediate step in our decision making process: we manually examined the Manhattan plots for many of the 161 genes with >10 reported peaks and found that there were frequently revealed strong and scattered signals across the genome. The population structure and other confounding factors were simply speculation as to why the GWAS analysis failed in this subset of cases. We have revised our text to explain the actual reasoning and evidence for our decision making rather than our speculation about the reason behind the pattern we observed (Page 6 Line 1-4).

Page 7, lines 24-25, not clear why 0.33 is modest, it is still in disequilibrium, probably you can delete "modestly linked" and substitute it with "in disequilibrium"

Thank you for pointing out the issue with the terminology here. We have now changed this sentence to read “Despite being located only 7.4 kb apart, the cis-eQTL and cis-sQTL located within Cys2 have a disequilibrium r2 of 0.33 suggesting that these two SNP markers may define separate functional variants controlling expression levels and transcript splicing (Figure3f).”

Page 7, line 60, substitute "WE" with "We"

Corrected

Page 12, line 11, substitute "Parviglumis" with "parviglumis"

Corrected

Page 13, line 33, substitute "temperat" with "temperate"

Corrected

Table S1, sometimes you use P for statistical significance (in the text) but here you use p, like in this Table. I suggest that you use pin the whole manuscript

Thank you for the suggestion, we used lower case p to indicate p value throughout the revised manuscript.

Reviewer #2: The authors identified the variations in maize gene expression and alternative splicing, by generating 572 RNA-seq datasets from the roots of 340 maize individuals. They identified numbers of eQTLs and sQTLs based on association analyses, and estimated the differences in the broad-sense heritability between the tropical and temperate maize groups. The whole project is well designed to include data repeats for reliable downstream analyses. The generated population scale RNA-seq data are good resources for the community. **However, the manuscript was not well organized, the logic in the "Results" and "Discussions" sections are confused, which make the main points and highlights unclear.** Please find below in more detail some major concerns as well as several minor issues.

Major

1. The whole manuscript is not well organized. The "Results" section is a mixture of "how we did these analyses" and descriptions of the basic distribution of the results, which from my point of view, the former part should be moved to the "Methods" section, while the later part should be curtailed and more biological-meaningful results should be added to highlight the main findings. Meanwhile, the "Discussion" section contains lots of conclusions that should be provided in the "Results" section.

For example, in Line 11-24 (Page 6 of 27), the authors spend lots of efforts to explain how the alternative splicing (AS) analyses were performed. To my knowledge, the AS were identified based on canonical PSI value based method with no special modifies, thus is needless to emphasis in the Results section.

Thank you for pointing this out. We have moved a great deal of the text dealing with alternative splicing analysis to the methods section (Page 17 Line 1-17) and focused and condensed the remaining discussion of alternative splicing within the results section (Page 6 Line 41-46; Page 7 Line 1-7),

In the "Expression quantitative trait loci reveal the genetic architecture of transcriptional regulation" sub-section (Page 5 - Page 6). The authors spend lots of works describing the results of eQTLs. There are mainly three results: i) identified 7,691 eQTLs including 7,105 cis-eQTLs and 586 trans-eQTLs; ii) there are no trans-eQTL hotspots and iii) trans-eQTLs represent more rare alleles.

However, there lack discussions on the importance of these findings and the possible reasons that causing these results (biological or statistical? Based on the biased proportions of cis and trans eQTLs, it is reasonable to doubt that the association between rare alleles and trans-eQTLs may be caused by statistical bias, say if rare alleles are more like to occur in regions distant to genes. Thus, I suggested the authors to perform some enrichment analyses to support their findings).

We apologize for not stating this point clearly. We identified many more than 586 trans-eQTLs but in only 586 cases was a trans-eQTL identified for a gene where no cis-eQTL was identified. In most cases where a trans-eQTL was identified cis-eQTL were also identified. We have revised the manuscript to avoid this same miscommunication/misunderstanding.

Based on feedback from you and other reviewers we have removed the discussion of rare alleles at trans-eQTL being associated with extremes of expression from this manuscript. This should, hopefully, shorten up this section of the manuscript and make it easier to follow.

Besides, I did not see the connections between these findings and the other sub-sections of the results, as well as the main point (as inferred from the title) of "a role for heritable transcriptomic variation in maize adaptation to temperate environments". I suggested the authors rearrange the Results and Discussion sections to simplify the descriptive statements, highlight the main points and how did them support the main findings of the study.

Based on the reviewer’s concerns we have made a number of modifications to our manuscript.

First, based on the concerns expressed by both you and reviewer #3 we have made the decision to remove the analysis and discussion of rare alleles associated with trans-eQTL as reviewer #3 raises important methodological concerns and this analysis is not central to the key findings of the paper.

Secondly, we have changed the title for the subsection previously titled “Expression quantitative trait loci reveal the genetic architecture of transcriptional regulation” to “Quantifying the heritable genetic control of transcriptional variation in maize” and shortened the text of this section.

Thirdly, we have shortened the description of eQTL and sQTL mapping and consolidated the results of these analyses into a single figure.

2. The authors identified genomics regions exhibiting significant selective sweep signals for adaptation to temperate environment, and listed several known gene cases that located in the select regions. However, the results lack the general genomic patterns of these select regions. The evidences of several gene cases are insufficient to support the sub-title (line 38 page 8 of 27) of "Genomic mechanisms underlying temperate adaptation". To my knowledge, the selection signals for maize adaptation to temp environment have already been studied by Hufford et al. (<https://urldefense.com/v3/__https://doi.org/10.1038/ng.2309__>;!!PvXuogZ4sRB2p-tU!BVWooBMemQgx84hL_QgEfNogOK9filp-EQS2Jq6wp0GNjZc6rIfJStd0wtM5JZ58IiY1-DfbmWGygHHr$ ) and Liu et al (<https://urldefense.com/v3/__https://doi.org/10.1016/j.molp.2015.01.016__>;!!PvXuogZ4sRB2p-tU!BVWooBMemQgx84hL_QgEfNogOK9filp-EQS2Jq6wp0GNjZc6rIfJStd0wtM5JZ58IiY1-DfbmWLH1ENX$ ), while the later used population level RNA-seq from kernels of a maize association mapping panel (AMP) including tropical and temperate individuals, similar to the population used in the current study. Besides. there are also public data of the RNA-seq from leaf in response to drought resistance

(<https://urldefense.com/v3/__https://doi.org/10.1186/s13059-020-02069-1__>;!!PvXuogZ4sRB2p-tU!BVWooBMemQgx84hL_QgEfNogOK9filp-EQS2Jq6wp0GNjZc6rIfJStd0wtM5JZ58IiY1-DfbmXgmw9mu$ ), population level metabolomics (<https://urldefense.com/v3/__https://doi.org/10.1038/ncomms4438__>;!!PvXuogZ4sRB2p-tU!BVWooBMemQgx84hL_QgEfNogOK9filp-EQS2Jq6wp0GNjZc6rIfJStd0wtM5JZ58IiY1-DfbmWp9pWz-$ ) and DNA methylations (<https://urldefense.com/v3/__https://doi.org/10.1186/s13059-019-1859-0__>;!!PvXuogZ4sRB2p-tU!BVWooBMemQgx84hL_QgEfNogOK9filp-EQS2Jq6wp0GNjZc6rIfJStd0wtM5JZ58IiY1-Dfbmcma-L6Q$ ) for the AMP population. Leveraging these multi-omics resources could help the authors draw more comprehensive conclusion on the temperate adaptation of maize. Why these resources were not considered in the authors' analyses? What are the new findings that the authors could bring to the readers with the new RNA-seq data from root than those from kernels and leaves in AMP population?

Response: Thank the reviewer for pointing out these resources. First, we agree with you that the previous title of this subsection overstated the strength of the results. We have revised the header for this section to read “Genomic regions associated with temperate adaptation in maize” which we feel is more consistent with the nature and strength of the results we discuss.

We also agree that there is value in the previous studies you cite that conducted analyses of selective sweeps and/or generated transcriptomic or multi-omic resources. One challenge to keep in mind is that the process of maize adaptation to different climates and agronomic practices has been quite complex. And different studies can identify very different sets of genes depending on the specific populations employed and the specific criteria used to sort maize lines into those categories.

Specifically, Hufford et al, a similar study we cited in our original manuscript but discuss more extensively in this revised paper, the focus was on 1) domestication, comparing wild teosinte to domesticated maize and 2) improvement, comparing maize landraces to modern elite inbreds. Because maize was domesticated in tropical southern Mexico and many breeding efforts today are based in temperate regions, a comparison of landrace and elite inbred maize will be imbalanced, with tropical lines overrepresented among landraces and temperate lines over represented among elite inbreds. However, this partial overlap still results in identifying a very different list of genes from the set identified when temperate vs tropical origin is the actual criteria used to divide lines into groups for analyses of changes in genetic diversity (see venn diagram below).

Liu et al focused more specifically on the question of maize temperate adaptation, like our study. However, even in this case, the set of genes identified as targets of selection shows only modest overlap (again see venn diagram below). Here are some explanations for the limited degree of overlap and why we felt it was important to run our own analyses rather than rely on the published gene list of Liu:

1) The first reason is the different maize materials.In Liu’s work the temperate maize was predominantly (75%) from China and the tropical lines were predominantly from Mexico. In our work, temperate lines were drawn primarily from the USA and tropical lines from both Mexico and Nigeria. As a result of different allele and haplotype frequencies in these different populations, different genes were identified as targets of selection. For example the widely studied maize improvement and adaptation genes yellow 1 (*y1*) (kernel composition), and the maize flowering-time adaptation gene, zea centroradialis8 (*zcn8*) (flowering time), both known to be targets of selection were identified in our study but not by Liu et al 2015.
2) Secondly, a key limitation of the Liu et al study that may partially explain the limited overlap between our gene sets is that their SNP set was generated from RNA-seq data and so focused only on exonic regions of genes, largely excluding signal from introns, promoters, and upstream regulatory sequences. Our subsequent analyses was able to benefit from widespread resequencing efforts to generate and employ a genome-wide SNP set capturing both exons, introns, promoters and non-genic regions. This was a key limitation acknowledged in the Liu et al paper -- “the features seem to be underestimated” in their study and “intergenic regions were considered as more diverse” -- but a necessary one given the technology and datasets available at the time.
3) Finally, a very pragmatic reason it was important to repeat the analysis is the poor translatability of gene models between genome versions. We employed the B73 RefGen_v4 genome, which is already out of date now, but which was the most recent version of the genome when we began work on the analyses published in this paper. Liu et al employed B73 RefGen_v2, which likely was also the most recent version of the genome at the time they commenced their analyses. However, only 1,341 out of 1,660 candidate genes identified by Liu in version 2 have equivalents in the B73 RefGen_v4 genome (20% lost). Similarly, only 1,571 of our 2,053 candidates have equivalent genes in B73 RefGen_v2. For example, *MFT* (Mother of FT and TFL1, GRMZM2G059358) was identified as a strong candidate gene in adaptation by Liu et al., (2015). However, this gene missing in both v4 and current v5 annotations, and no sequence homology was found in the syntenic region of other cereal plants (sorghum, foxtail millet, rice and brachypodium)	(<https://www.maizegdb.org/gene_center/gene/GRMZM2G059358>).

In any case, in our revised manuscript we now cite and discuss Liu et al, its significant contributions to the field, and the reasoning behind our decision to conduct an independent analysis of genes that experienced significant losses of diversity during domestication rather than reuse the Liu et al 2015 gene list in our own study.

We also cite and discuss Liu et al 2020’s very nice paper on eQTL identification across well watered and drought stressed conditions.

We regret that the scope of our paper does not extend to metabolomic analysis as described in Wen at al 2014, which likely act as intermediates between gene expression and whole plant phenotypes (measured in this study) nor did we have space to include discussion or analysis of the role epigenetic modifications (including DNA methylation as described in Xu et al 2019) play in mediating DNA sequence variation in both cis and trans with changes in gene expression level. But we agree both of these are important and possibly essential lines of investigation in the ultimate effort to link genotype to phenotype and now discuss these papers in our revised introduction.

**Reviewer Response Fig. 1: Maize genes identified as targets of selection in different studies and contrasts**. Dom, genes for maize domestication identified by Hufford et al. (2012); Imp, genes for maize improvement identified by Hufford et al. (2012); Adap, genes for maize adaption identified by Liu et al., (2015); This, genes for maize temperate adaption identified in this study. All gene model IDs from maize annotation B73 RefGene_v1 (Hufford et al 2012) or v2 (Liu et al 2015) were translated to B73 RefGen_ v4 IDs (This study) using the translation table provided by MaizeGDB (<https://www.maizegdb.org/gene_center/gene#translate>). This figure is now included as supplemental Figure S9.

3. The authors discussed the differences between broad sense heritability and narrow sense heritability in the Discussion section (Line 42-49 Page 10 of 27). However, only the broad sense heritability was estimated in the authors results. The authors could also perform analyses on the narrow sense heritability differences between tropical and temperate maize, to draw more solid conclusions.

Thank you for making this point. In our revised manuscript we include a new analysis comparing broad sense estimates of gene expression heritability derived from observations of multiple individuals with identical genotype to narrow sense heritability estimated from the kinship matrix and shared relationships inferred among different genotypes as Figures S1A & S1B. The relationship between these two variables is reasonably strong, with our approach to estimating broad sense heritability exhibiting modestly superior dynamic range (an estimated narrow sense heritability of 0 corresponds roughly to an estimated broad sense heritability of 0.1 and an estimated narrow sense heritability of 1 corresponds roughly to an estimated broad sense heritability of 0.75).

In practice the issues of dominance and epistasis being captured by broad sense heritability are most in inbred individuals and very small in measured effect size, respectively so we suspect this improved dynamic range is a result of different calculation method it was possible to use for broad sense heritability of gene expression rather than representing fundamental difference between the theoretical types of heritability being measured.

4. In Line 11-27 (Page 7 of 27), the authors illustrated two cases for the colocalization of eQTLs and sQTLs (also showed in figure 3e and f). I suggest the authors to dig deeper into the detailed causations of these colocalizations. Is the leading variant the causal variant? Does the expression and splicing variations caused by the same causal variation? Or at least, the authors could provide additional information in figure 3 to illustrate how did the expression and splicing alter along with different alleles.

Thank you for the suggestion. In general, we could not confidently conclude that the lead SNP was the causal polymorphism without additional lines of evidence. We have added additional discussion of these two cases. In the case of CT2, it seems likely that a single variant is responsible for both the eQTL and sQTL signals. In the case of Cys2 we see only limited correlation between the sQTL and eQTL suggesting these are likely two distinct functional variants. Based on your feedback we had added two panels to the relevant figure and added more text to discuss this point.

**Original question:** 5. The authors estimated the H2 for gene expressions and perform ICA for AS. What is the H2 for AS between tropical and temperate maize? Can ICA and ICs-based GWAS be applied to gene expressions?

**Our interpretation of the question, please correct if we are wrong:** 5. The authors estimated the H2 for gene expressions and perform ICA for gene expression. What is the H2 for AS between tropical and temperate maize? Can ICA and ICs-based GWAS be applied to AS?

Response: Thank you for this suggestion. In this revised manuscript we include an analysis of the heritability of alternative splicing patterns measured in this manuscript and differences in pattern between tropical and temperate populations similar to the analyses included for gene expression in the previous version.

Unlike overall gene expression level, alternative splicing shows a very skewed distribution with a large number of events exhibiting estimated broad sense heritability values near zero (see below). Estimates of the heritability of intron level AS in temperature germplasm were more likely to be in the interval from 0.10-0.25 and less likely to be in the 0-0.1 and 0.25-1.00 windows than estimates from tropical germplasms (see below).

A key assumption of ICA analysis is that each signal is a combination of multiple independent linear relationships. This assumption is violated in the case of AS data where multiple PSI values calculated from the same gene are not independent of each other. While it might be possible to control for this in some way via subsampling of the data, it would require substantial new methods developing/validation that is not central to the core conclusions of our paper. Given this concern, as well as the low heritability of the majority of intron-level AS measurements we did not proceed to ICA and IC-based GWAS for these features but we now discuss the results from the new analysis proposed by the reviewer in our revised manuscript and include the panels below as Figures S1C and Figure S1D.

**RR Figure 2: Overall distribution of estimated broad sense heritability of intron PSI values** (e.g. alternative splicing) on the left, and relative distributions when AS heritability is calculated separately for tropical and temperate germplasm.

Minor

1. There are several typos that should be avoided, to list a few

Line 44 (page 5 of 27): "... was typically lower the estimate of total variance explained ...";

Corrected

Line 61 (page 7 of 27): "WE";

Corrected

Line 51 (Page 8 of 27): "exhibitED";

Corrected

Line 8 (Page 12 of 27): "showeing";

Corrected

2. All the figure legends were not well organized. The basic representations of each sub-figure or track should be added, rather than directly using a conclusion of what the figure meant to the readers as the sub-title. For example,

Figure 1 "(d) Distribution of broad sense heritability of genes in the tropical and temperate panels;", what's the meaning of the red dash line?

Figure 2 "(e) Rare trans-eQTLs are associated with extremes in gene expression, as identified in the entire population.", this is more of a conclusion than a figure legend.

Figure 5 is not cited in the article, and some panels in Figure 6 are not cited.

I suggested the authors to review and re-organize all the legends to make them more informative.

We have revised these figure legends to include more detail and ensure all panels are cited in the main text.

3. Line 6-8 (Page 6 of 27): "These results are consistent with disregulation of expression induced by trans sites imposing similar fitness burdens as those previously observed at cis-regulatory sites." Please add references that support this conclusion.

We have removed this analysis from this revised manuscript, therefore have also deleted this conclusion.

4. Figure 6a contains too many points to be provided as vectogram. My pdf viewer spends extra time loading the paths of these points each time I navigated to this figure. Please reduce the paths in the vectogram or make it a bitmap image.

We apologize for the inconvenience this created for the reviewer. In this revised manuscript we converted the original svg/pdf versions of this Figure to a jpg before compiling our final PDF for submission. We have also converted a number of other scatterplots to 2D kernel density plots which both reduces load times and often makes it easier to spot patterns in distributions with tens of thousands of individual datapoints. Hopefully this will fix the issue.

5. Line 41-42 (Page 7 of 27): what is the total variance explained by the retained 43 ICs?

We calculated the variance of each IC across the population and the sum of the variance explained by the 42 retained ICs (we re-analyzed these data and now only 42 ICs) was 0.3414759. This information has been added to the revised manuscript (Page 8 Line 12-14).

Reviewer #3:	The authors present and analysis of a large and high quality transcriptome dataset from diverse maize genotypes. The analysis focuses on identifying QTLs for expression and splicing, partitioning those between cis and trans, while studying whether the genetic architecture of expression has changed during the evolution of temperate lines from tropical lines. They show evidence of changes in the heritability of certain classes of genes, identify several interesting candidates with potential contributions to temperate adaptation, study the co-localization of expression and splicing QTL, and study the hypothesis of rare allele burden on expression.

Overall, I think the dataset is strong, and the analysis is well done. I particularly like the ICA analysis and think it's a power approach to these data. I do have some trouble following the main story of the paper - is it a study of temperate adaptation or a study of the architecture of expression variation? If it is both, I think the link between them can be made more explicit. I also have a major concern about the trans-eQTL results which I'll discuss below, and several questions and more minor comments on the presentation.

My major concern with the results is that I'm not convinced the trans-eQTL results are robust - they are likely dominated by false positives. My evidence for this is:

1. Figure 2c shows that the detected trans-eQTL explain very little variation. With a sample size of only 340 lines, the power to detect a QTL that explains 5% of the variation with a threshold of 4.1e-9 is only about 4%. This would mean that there should be 25x as many trans-eQTLs of similar magnitude to your discoveries in this population, which would be a very large number

We agree that with a sample size of 340 lines, the power of detecting trans-eQTLs is not high which means a substantial portion of trans-eQTLs with small effects were not captured. This is the motivation of performing ICA to capture signals from genomic intervals which have modest effects on the expression of large numbers of genes.

We have conducted additional permutation tests to estimate false discovery rates via a different mechanism in this revised version of the manuscript (described in more detail below).

2. Figure 2d shows that these detected trans-eQTLs explain little variation because the markers are very rare (modal maf < 0.1). This means that most of these trans-eQTLs are represented by only a few individuals in the dataset. It looks like you used a maf cutoff of 0.01 for the eQTL, meaning that you'd be testing markers with only 4 individuals represented by the minor allele. The problem with this is that the linear models get very sensitive to departures from normality of residuals when one of the alleles is rare (we lose the guarantees of the central limit theorem). The box-cox transform helps, but isn't flexible enough to ensure that residuals will be normal. You could try more extreme normalizations like rank-transformation or quantile-normalization. Genotype permutations could be used to test whether rare alleles are likely to give false positives - stratify the permutations by allele frequency and look at the false positive rate separately for maf < 0.05 and >

0.05; care is needed to ensure the permutations don't break population structure-phenotype associations. But the safest would be to simply throw out markers with maf < 0.05 (or maybe 0.1), perhaps guided by genotype permutations.

Thank you for the intuitive comments. We absolutely agree that a great deal of caution is necessary when testing for associations involved in rare alleles, particularly in a population of only 340 distinct genotypes.

However, the situation is not quite as dire as it likely appeared from the first version of the manuscript. We were not as clear as we should have been in our methods section. While a criteria of minor allele frequency > 0.01 was used for the initial filtering of unimputed SNPs, the dataset was filtered a second time with the criteria MAF > 0.05 after imputation. All results presented in our manuscript are from a set of approximately 12 million markers with a MAF > 0.05. We have revised our manuscript to more clearly explain the filtering criteria employed and the characteristics of the final SNP set.

As suggested, we performed a permutation based strategy to calculate the possibility a SNP is identified as an eQTL by chance. After permuting the labels used to connect the marker data and the gene expression data, MatrixEQTL was used to conduct eQTL analysis for all genes. Among the maximum -log10(p-value) observed for each gene, the 95th percentile corresponded to a -log10(p value) of 7.89. This is notably less stringent than the bonferroni corrected cutoff of -log10(p-value) of 8.39 used throughout the manuscript. Bonferroni correction is almost certainly excessively conservative as it assumes independence between each genetic marker and we observe substantial LD among the marker dataset used in our study. In addition, for all genes where the maximum -log10(p-value) obtained from permuted data was above the 95th percentile, the peak SNP was located in trans relative to the target gene rather than in cis, suggesting that, consistent with the reviewer’s suggestion, the cutoff for cis-eQTL discovery could likely be relaxed relative to trans-eQTL if we calibrated these two cutoffs separately, or alternatively that the false discovery rate among cis-eQTLs is likely substantially lower than among trans-eQTLs. However, our approach to permutation did not preserve the greater phenotypic similarity expected among genotypes with closer relatedness as inferred from the kinship matrix so we did not feel comfortable using this result to lower our p-value thresholds. We’d propose to present our results with the original more stringent threshold, although we are happy to repeat the permutation test if you are aware of a more appropriate way to permute the data that more accurately preserves kinship patterns.

Also, note that MatrixEQTL doesn't fit a mixed model, unlike the more complete GEMMA model used for other traits, and 5 PCs are less likely to pick up subtle relatedness that may generate correlations among individuals at rare haplotypes. You might try seeing if some of these rare trans-eQTLs remain significant if you fit them with GEMMA or some other full LMM.

Based on your feedback we conducted parallel analyses for a set of 3,000 genes using both MatrixEQTL and GEMMA. The overall distribution of eQTLs per gene were similar, as are the distribution of minor allele frequencies for cis-eQTL. However, we do observe a 20-30% enrichment if the frequency of trans-eQTL between 0.05-0.1 MAF in the MatrixEQTL results relative to the trans-eQTLs identified by GEMMA. In the revised manuscript we include this new comparison as supplemental Figure S4, discuss the potential elevated false positive rate for trans-eQTL with MAFs between 0.05-0.10 (Page 6 Line 29-39) and have revised our figure design to provide separate counts of the number of trans-eQTL excluding those between 0.05-0.10 (Figure S3F).

**RR Figure 3:** **Comparison of eQTLs identified by GEMMA and matrixEQTL for a subset of 3,000 genes**. (a) Number of genes with 0, 1 and more than 1 eQTLs identified by GEMMA and MatrixEQTL.(b) Minor allele frequency of *cis*-eQTL and *trans*-eQTL identified by GEMMA and MatrixEQTL. (c) Different number of peaks identified by GEMMA and MatrixEQTL. Manhattan plots is generated based on the p-values from Mixed Linear Model (MLM) implemented in GEMMA for gene Zm00001d000501. GEMMA identified only one eQTL on chromosome 3 at the threshold 4.10e-9 (blue dashed horizontal line), while MatrixEQTL identified four (red vertical lines).

3. Figure 2c/d shows that in contrast cis-eQTLs are not found with low PVE or low MAF.

If you could accurately detect trans-eQTLs at maf<0.05, or pve < 0.05, why couldn't you similarly detect cis-eQTLs of the same effect sizes?

These were poorly designed graphs (more on this below) and we sincerely apologize for them. We did not detect any trans or cis-eQTL with minor allele frequencies less than 0.05 (as SNPs with MAF < 0.05 were not including in our marker set for GWAS), but plotted a 1D kernel density plot with an x-axis that extended beyond the minimum value present in the distribution. We detected 119 cis-eQTL with PVE < 0.05.

We have revised the original Figure 2D (now Figure S3f) so the x-axis stops at 0.05 to avoid the previous miscommunication of results. Figure 2C is now Figure S3e.

The simplest explanation is that the proportion of cis-SNPs that have large effects is much greater (orders of magnitude greater!) than the number of trans-SNPs that have large effects. For cis-SNPs, if you have a false positive in 1/1e7 SNPs, but true positives in 1/1e4 SNPs, then your positives are dominated by true positives. But the number of SNPs-gene combinations that is a true trans-eQTL is probably much lower (say 1/1e8). In this case, your positives are dominated by false positives. One potential solution could be to calibrate significance thresholds separately for trans- and cis- eQTLs, or to use false discovery rates to select hits separately for trans- and cis- eQTL

We agree with you that the proportion of trans-SNPs that have large effects is almost certainly much smaller than the proportion of cis-SNPs that have large effects. However, our rate of false positives appears to be substantially lower than 1/1e7 at least based on the results of the permutation test described in our answer to your question #2 above. The idea of calibrating separate significance or false discovery thresholds for trans- and cis- eQTLs makes a lot of sense and we would like to explore this idea further. In this case the thresholds for both cis and trans eQTLs appear to fall below the extremely conservative bonferroni corrected p-value appear used in this paper (see caveat in our answer to question #2 about whether there is a better way to perform permutations in this case.

4. Figure 2e shows that for trans-eQTL pairs, the number of rare alleles correlates with expression rank. This is similar to the analysis in the Kremling paper, but subtly different in important ways. Here, you're only including trans-eQTL pairs that you detect as significant. Figure 2d shows that for the vast majority of these the maf is in the "rare" category. The fact that this is an eQTL means that individuals that carry the rare allele have either higher or lower expression than individuals that carry the common allele. And individuals that carry the rare allele probably share a rare haplotypes that also has other rare alleles as well. So this is really a circular argument. The Kremling analysis was not circular because all cis-eQTL pairs were included whether or not they were "significant" in association analyses. That's impossible to do here because you would have to compare the ranked expression to rare allele counts across all windows of the genome which wouldn't

be informative. Also, why was a 0.2 threshold used for "rare"? That's not very rare. Kremling used 0.05. I'll note that Figure 2f is hopeful in that it's hard to see why the smile would replicate in a different sample if it was driven by false associations. But it is possible that shared population structure between the two data partitions could lead to this (eg. Josephs et al <https://urldefense.com/v3/__https://doi.org/10.1534/genetics.118.301786__;!!PvXuogZ4sRB2p-tU!BVWooBMemQgx84hL_QgEfNogOK9filp-EQS2Jq6wp0GNjZc6rIfJStd0wtM5JZ58IiY1-DfbmbjEpBlV$> )

In summary, I think more is needed to claim that the trans-eQTLs, and the rare allele burden results are more than just noise.

This is also a major concern from reviewer 2. Upon reflection on the points you brought up here, we agree with you that there are significant flaws/circularities in this analysis. Since this analysis was not essential to the major findings and conclusions drawn in this study and, as you say, requires significant additional evidence to support its conclusion, we have removed it from this revised version of the manuscript.

On the question of paper focus, I think the idea of looking for selective sweeps at the genomic and expression level is interesting, but could be developed further. I think the hypothesis here should be made more explicit, especially in the introduction. Lines 33-36 on page 10 describe one reason for a change in heritability. But there are other explanations including changes in genetic architecture due to drift or selection. If the frequency of functional variants decreases, Vg will decrease, decreasing h2 even if the environmental responsiveness is constant.

Thank you for the comment, this is a very good point, we now discussed the additional potential explanations in the revised manuscript (Page 11 Line 23-27)

More minor comments and concerns:

- GWAS methods:

- how were replicates dealt with in eQTL analysis?

Thank you for pointing out the lack of detail at this point. We have added the following statement to our methods section:

“eQTL mapping was conducted using MatrixEQTL(v2.3). For each distinct genotype, average values across biological replicates were calculated. For each gene, expression values were transformed using the Box-Cox method prior to mapping.”

- it looks like in ICA replicates were included as samples, and then collapsed to BLUPs afterwards

This is correct for the ICA analysis specifically.

- what about for individual gene expression traits? It looks like 2 seeds were grown per tube. Were these the two replicates, or were 2 tubes grown per line? If the former, these 2 seeds cannot be used to calculate broad-sense heritability because they share more than just genotype, also environment.

We apologize for the lack of clarity in the methods section of the previous version of the manuscript. Tissue from kernels grown together in the same tube were pooled for RNA extraction and subsequent RNA-seq/gene expression quantification. Replicates in this study consisted of sets of kernels from the same maize line grown at different times. We have revised our methods section to reduce the risk of confusion regarding this point.

- Fig 2a - The PVE of an eQTL is likely to be over-estimated unless you re-estimate the effect size in a new dataset. This is worth mentioning and can explain why some values are above the diagonal

Yes, this is an important point to bring up. We have added the discussion of this observation in the revised manuscript (Page 6 Line 13-17): “It should be noted that percent variance explained values presented in this study were calculated from the same dataset used to identify eQTLs. As a result, these values are likely to be inflated to some extent relative to the percent variance explained that would be observed for the same eQTLs in an independent dataset.”

- Are the ICA SNPs in genes loading on the ICA axes? p8.top - by snps being "linked" to genes, I think you mean the snp is physically located in or nearby this gene, not as an eQTL, right?

Yes, you are right these are genes linked to a given IC based on proximity to SNPs identified on a GWAS analysis for IC values, rather than genes which contributed to the ICs. That specific sentence was removed in this revision but we’ve also added more detail on how candidate genes were linked to individual ICs to the methods section.

- Fig 7: what is the vertical blue bar in the left-hand panels?

They are the indications of the genes described in those panels. Thank you for pointing out this omission. We have revised the figure legend to include this information (Now Figure 5 in the revised manuscript).

- Fig S5 - what is the blue vs grey lines? Are you showing ICA loadings for all genes here or only ones significantly associated with an IC?

The blue line was the overall IC while the gray lines were the individual genes significantly associated with the IC. Based on your feedback and our internal discussions we decided this way of visualizing ICs was not particularly helpful. The revised visualization is shown in Figure S7, panels B, C, and D. We have also expanded the legend of Figure S7 to provide more detail on the visualizations.

- Figure 1d-e and 4.57. To ask whether H2 was lower in temperate vs tropical maize lines, you should use a paired t-test, or equivalently do a one-sample t-test on the difference between H2 values per gene. The regression slope doesn't give this. The R2 of the regression would be equivalent to the correlation between H2 values which could be interesting as well. Also, 1e is sort of a trivial result - of course the blue density is shifted left than the red density - genes with higher values of H2-tropical will tend to have values of H2-temperate lower than H2-tropical almost by definition. The only interesting addition to 1d I could imagine would be to show the densities of H2-temperate and H2-tropical along the margins of 1d.

You are right. We have removed the regression analysis from the revised manuscript. When performing a t-test based analyses we actually observed the opposite pattern with a modest but very statistically significant trend towards higher heritability among temperate genotypes relative to tropical. This new result is included in our revised manuscript. Thank you!

**Second round of review**

**Reviewer 1**

The authors have provided a much clearer manuscript, thank you, it was a pleasure to review

**Reviewer 2**

The authors have addressed all my concerns and i have no more questions! I am wishing to see the final publication.

**Reviewer 3**

The authors have addressed most of my concerns from the first submission. I appreciate the thoughtfulness and care with which they addressed these concerns. I have several follow-up comments:

Overall, I think the cis- eQTL and sQTL analyses are really interesting, especially the relationship between eQTL and sQTL. The IC mapping also has interesting and high quality results. However, I still have concerns about the trans-eQTLs:
- The permutation test does increase my confidence that many of the discovered trans-eQTLs are actually true positives. It would be helpful to more carefully describe the permutation test in the methods section. The authors concern about using the permutation-based threshold because of breaking the association between the phenotypes and the kinship is valid. I'm not sure if they did it this way, buy the best way I know of doing this is to permute the genotypes but keep the PCs un-permuted. This way the PCs explain the same amount of variance under the permutations and only the tested marker changes. It's still not perfect, but better than permuting the phenotypes which breaks both the association with the markers and with the kinship. But since the permutations (at least as currently done) suggest a more stringent threshold, I'd agree with the authors to keep using the bonferroni threshold.
- However, even if the trans-eQTLs are "true", the result remains that they (individually) explain a vanishingly small amount of the phenotypic variance, relative to cis-eQTLs. Given this, and the previously discussed issue with the power to detect small-effect QTL in this population, I'd recommend still de-emphasizing many of the results based on the discovered trans-eQTL. For example, the observation of a lack of trans-eQTL hotspots is likely a power issue. If you only have ~4% power to detect a QTL explaining 5% of Vp, and if this is towards the upper-bound of most trans-eQTL effect sizes, then you're likely missing >95% of the e-traits regulated by each trans-locus which is why you don't see the trans bands. The contrast with bi-parental families is that in those the trans-loci are at MAF = 0.5, so you have much more power to detect them. Similarly, any statistic where you count the number of trans-eQTL should be made more explicit that these are the "detected/discovered" trans-eQTL. For example, line 6.5-8, the 45.8% of e-traits with 2+ eQTL having a trans-eQTL is certainly a vast underestimate of the number of these e-traits that have any trans locus that explains a non-zero amount of their variances.
- I think the same concerns applied equally (or more) to the sQTLs as well.
- I can't find the methods describing how eQTL peaks were described. Are you doing a clumping procedure based on physical distance and/or LD? See line: 8.27: "distinct peaks as described above". Line 6.5-6 suggests only a single cis-eQTL can be discovered. Why is this? Couldn't there be multiple cis-associations as long as the lead SNPs are not in LD with each other?

Other questions/concerns:
- 5.4 - it looks like the variance of h2 is greater than H2. The regression line doesn't really seem to capture this relationship. Do you get the same result if you switch the axes?
- Fig S1b. How can h2 > H2? This doesn't seem to be mathematically possible is h2 = H2 - s2_{non_additive_G}/s2_P. I think this might be because s2_P is different between the two axes - on the y-axis, s2_P is for the mean expression across genotypes, while for the x-axis, s2_P is for the variance in expression across samples.
- 8.17-21: "This observation suggests the latent features identified by this method might represent trans-eQTL hubs composed of a large number of small effect regulatory genomic loci that accounted for the heritability could not be captured by traditional eQTL mapping. " I don't understand this statement
- 8.27-28: "A peak represented by the lead SNP located at 28141 Mb on chromosome 9 (9:141,083,854) was associated with variation in the expression of 26 out of 59 genes in IC39 module with an enrichment p of 9.8e-68". I'm not sure I follow this. I thought the GWAS was on IC39. Did you follow that up by also looking at the separate eGWASs of each of the 59 genes in IC39? Did you do that for all the lead SNPs for each of the IC modules?
- 9.1-5: "the e-traits of this peak identified by GWAS included many genes associated with plant pathogen responsive genes such ... These observations are consistent with the functions enriched in the module associated with IC101 (Fig. 3d)" Again, is this based on the eGWAS eQTL results based on the SNP-IC101 associations? It seems circular. If this peak is associated with IC101, and IC101 contains plant-pathogen responsive genes, then of course this peak is associated with the expression variation in plant-pathogen responsive genes.
- 9.7-11: "Obvious differences in the expression level of several genes within clusters was observed for several of the genes in modules associated with IC39 (Figure S7b) and IC79 (Figure S7c), but no obvious differences were observed in genes from the co-expression module associated IC101 (Figure S7d)." What "obvious" differences are referred to here? I can't really see any differences by eye. The mean expression looks pretty consistent between populations. There are some genes that drop out in some individuals in each population, but that looks gene-specific, not any property of the module.

**Authors’ response to reviewers**

Reviewer #1: The authors have provided a much clearer manuscript, thank you, it was a pleasure to review

Thank you for your time reviewing our manuscript, we sincerely appreciate that.

Reviewer #2: The authors have addressed all my concerns and i have no more questions! I am wishing to see the final publication.

Thank you for your time reviewing our manuscript, we sincerely appreciate that.

Reviewer #3: The authors have addressed most of my concerns from the first submission. I appreciate the thoughtfulness and care with which they addressed these concerns. I have several follow-up comments:

Overall, I think the cis- eQTL and sQTL analyses are really interesting, especially the relationship between eQTL and sQTL.

The IC mapping also has interesting and high quality results.

However, I still have concerns about the trans-eQTLs:

- The permutation test does increase my confidence that many of the discovered trans-eQTLs are actually true positives. It would be helpful to more carefully describe the permutation test in the methods section. The authors concern about using the permutation-based threshold because of breaking the association between the phenotypes and the kinship is valid. I'm not sure if they did it this way, buy the best way I know of doing this is to permute the genotypes but keep the PCs un-permuted. This way the PCs explain the same amount of variance under the permutations and only the tested marker changes. It's still not perfect, but better than permuting the phenotypes which breaks both the association with the markers and with the kinship. But since the permutations (at least as currently done) suggest a more stringent threshold, I'd agree with the authors to keep using the bonferroni threshold.

We appreciate your guidance on the best way to preserve population structure in permutation testing of GWAS results and we agree this is a more conceptually sounds approach to permutation.

We repeated our analysis permuting genetic marker data while keeping both phenotype (eTrait) and population structure PCs up-permuted. This results in a trans-eQTL discovery threshold of -log10(P) = 7.83 (alpha = 0.05), comparable to the threshold obtained in the previous permutation approach (-log10(P) = 7.89) and lower than the current threshold based on multiple testing correction (-log10(P) = 8.39). We speculate that this modest decline in threshold results from better controlling for false positive SNP associations resulting from population structure. Give a p-value of 10e-8.39, the results of permutation testing suggest a false discovery rate of 0.015 for trans-eQTLs.

We have updated Figure S3g to show the results we obtained using this method and updated the description of this analysis in the revised manuscript (p6 lines 30-35).. We have added permutation analysis into the methods (p17 lines 19-29)

- However, even if the trans-eQTLs are "true", the result remains that they (individually) explain a vanishingly small amount of the phenotypic variance, relative to cis-eQTLs. Given this, and the previously discussed issue with the power to detect small-effect QTL in this population, I'd recommend still de-emphasizing many of the results based on the discovered trans-eQTL. For example, the observation of a lack of trans-eQTL hotspots is likely a power issue. If you only have ~4% power to detect a QTL explaining 5% of Vp, and if this is towards the upper-bound of most trans-eQTL effect sizes, then you're likely missing >95% of the e-traits regulated by each trans-locus which is why you don't see the trans bands. The contrast with bi-parental families is that in those the trans-loci are at MAF = 0.5, so you have much more power to detect them.

We have rephrased the sentences describing the trans-eQTL statistics to read: “Considering e-traits with two or more identified eQTLs (but, as a result of the peak grouping strategy employed here a maximum of one possible cis-eQTL per e-trait), the overall breakdown was 54.2% of cis-eQTLs and 45.8% of trans-eQTLs and for a set of 3,521 e-traits with cis-eQTLs, trans-eQTLs were also detected (Figure S3c). Given the limited size of this mapping population and many trans-eQTLs exhibiting minor allele frequencies near 0.1, our power to detect trans-eQTL was necessarily limited and so the total number of true trans

regulatory variants is likely substantially higher than the number of detected trans regulatory variants. Likely as a consequence, we observed no obvious trans-eQTL hotspots, in contrast to a previous eQTL mapping study conducted in maize using a bi-parental RIL population [8] where both alleles are of functionally distinct regulatory variants are typically present in approximately 50% of individuals (Figure S3d).” (p6 lines 5-16)

Similarly, any statistic where you count the number of trans-eQTL should be made more explicit that these are the "detected/discovered" trans-eQTL. For example, line 6.5-8, the 45.8% of e-traits with 2+ eQTL having a trans-eQTL is certainly a vast underestimate of the number of these e-traits that have any trans locus that explains a non-zero amount of their variances.

We have added the qualification ‘detected or discovered’ throughout the paper when describing trans-eQTLs and trans-sQTLs.

- I think the same concerns applied equally (or more) to the sQTLs as well.

We have added similar qualifying text when describing the number of **detected** sQTLs.

- I can't find the methods describing how eQTL peaks were described. Are you doing a clumping procedure based on physical distance and/or LD? See line: 8.27: "distinct peaks as described above". Line 6.5-6 suggests only a single cis-eQTL can be discovered. Why is this? Couldn't there be multiple cis-associations as long as the lead SNPs are not in LD with each other?

1) We apologize for the omission. We have added a more detailed description of the approach we employed for eQTL peak grouping to the methods section of this manuscript. (p8 lines 32-35; p17 lines 12-17)
2) We have rephrased the results to refer more explicitly to the methods for information on the peak grouping approach. (p6 lines 5-7)
3) Briefly the approach we used employed physical distance with a very conservative (e.g. large) window size to avoid double counting signal from the same causal variants, however this means at most one cis-eQTL peak will be detected per gene. We have rephrases the text to make it clear this is a limitation of our method and not an assertion of a biological reality (we agree multiple independent cis regulatory variants can exist for the same gene, we’re just not confident we can distinguish them with this dataset).

Other questions/concerns:

- 5.4 - it looks like the variance of h2 is greater than H2. The regression line doesn't really seem to capture this relationship. Do you get the same result if you switch the axes?

We switched the axes and you were right that the regression line was not accurately capturing the relationship between the two variables. In this revision we have removed the regression line and added a simple x=y line (see below). Thank you for pointing this out!

- Fig S1b. How can h2 > H2? This doesn't seem to be mathematically possible is h2 = H2 - s2_{non_additive_G}/s2_P. I think this might be because s2_P is different between the two axes - on the y-axis, s2_P is for the mean expression across genotypes, while for the x-axis, s2_P is for the variance in expression across samples.

Thank you for pointing this out. You are correct that these are estimating broad and narrow sense heritability for different things (per sample expression values and per genotype expression values respectively). We have updated the figure legend to explicitly call out the distinction between these two.

- 8.17-21: "This observation suggests the latent features identified by this method might represent trans-eQTL hubs composed of a large number of small effect regulatory genomic loci that accounted for the heritability could not be captured by traditional eQTL mapping. " I don't understand this statement

Sorry about this confusion, there was a missing word in the previous version of this sentence. In this revised version we have split the sentence into two shorter ones:

"This observation suggests the latent features identified by this method might represent trans-eQTL hubs composed of a large number of small effect regulatory genomic loci. If so, these may explain a portion of heritability not captured by traditional eQTL mapping.” (p8 25-28)

- 8.27-28: "A peak represented by the lead SNP located at 28141 Mb on chromosome 9 (9:141,083,854) was associated with variation in the expression of 26 out of 59 genes in IC39 module with an enrichment p of 9.8e-68". I'm not sure I follow this. I thought the GWAS was on IC39. Did you follow that up by also looking at the separate eGWASs of each of the 59 genes in IC39? Did you do that for all the lead SNPs for each of the IC modules?

We apologize that this was unclear in the previous version of the manuscript. Yes, we did indeed conduct a subsequent follow up analysis examining individual genes whose expression associated with peak SNPs for each IC module where one or more GWAS peaks were identified for the values of the IC itself. We have reworded this sentence to make our intended meaning more clear: “We conducted genome-wide association study for each of the 42 independent components, followed by grouping of individually significant trait associated SNPs into distinct peaks as described above. Expression QTL mapping was conducted between the SNPs in IC GWAS peaks and expression of the 19,565 using a relaxed significant threshold (10e-5) for eQTL identification [49]” (p8 lines 33-37)

- 9.1-5: "the e-traits of this peak identified by GWAS included many genes associated with plant pathogen responsive genes such ... These observations are consistent with the functions enriched in the module associated with IC101 (Fig. 3d)" Again, is this based on the eGWAS eQTL results based on the SNP-IC101 associations? It seems circular. If this peak is associated with IC101, and IC101 contains plant-pathogen responsive genes, then of course this peak is associated with the expression variation in plant-pathogen responsive genes.

Part of the issue here is the same confusion as above, the result presented here is from a follow up analysis identifying individual genes whose expression was also associated with the peak SNP of the GWAS hit for IC101 itself. We agree that the individual genes whose expression is associated with the peak SNP of the IC101 GWAS hit being enriched in the same functions as the total set of genes included with IC101 is not a surprising result, but we felt it was an importance additional test to run, as the opposite result is not impossible and would have substantially shifted out interpretation of this result. We have revised this sentence to make it more clear that the result presented here is 1) the result of follow on gene level eGWAS results rather than the GWAS for IC101 itself and 2) not surprising or unexpected. (p9 lines 15-21)

- 9.7-11: "Obvious differences in the expression level of several genes within clusters was observed for several of the genes in modules associated with IC39 (Figure S7b) and IC79 (Figure S7c), but no obvious differences were observed in genes from the co-expression module associated IC101 (Figure S7d)." What "obvious" differences are referred to here? I can't really see any differences by eye. The mean expression looks pretty consistent between populations. There are some genes that drop out in some individuals in each population, but that looks gene-specific, not any property of the module.

Thank you pointing this out, this is an oversight on our end. We have reworded this sentence and it now reads ”The average expression of genes in IC39 (Figure S7b), IC79 (Figure S7c) and IC101 (Figure S7d) were responsibly consistent between tropical and temperate maize populations, although several individual exhibit large drops in IC39 and IC79. (p9 lines 23-26)
